# Targeting nicotinamide N-methyltransferase overcomes resistance to EGFR-TKI in non-small cell lung cancer cells

**DOI:** 10.1038/s41420-022-00966-x

**Published:** 2022-04-06

**Authors:** Jun Wang, Xi Liu, Yuanfeng Huang, Pan Li, Minqiang Yang, Shanshan Zeng, Danyang Chen, Qian Wang, Hao Liu, Kai Luo, Jin Deng

**Affiliations:** 1 Affiliated Cancer Hospital and Institute of Guangzhou Medical University, Key Laboratory of “Translational Medicine on Malignant Tumor Treatment,”, Guangzhou, Guangdong China; 2 Molecular Diagnosis Center, The Third Affiliated Hospital of Kunming Medical University, Kunming, Yunnan 650101 P. R. China

**Keywords:** Non-small-cell lung cancer, Mechanisms of disease

## Abstract

Activating mutations of epidermal growth factor receptor (EGFR) contributes to the progression of non-small cell lung cancer (NSCLC). EGFR tyrosine kinase inhibitor (TKI)-targeted therapy has become the standard treatment for NSCLC patients with EGFR-mutations. However, acquired resistance to these agents remains a major obstacle for managing NSCLC. Here, we investigated a novel strategy to overcome EGFR TKI resistance by targeting the nicotinamide N-methyltransferase (NNMT). Using iTRAQ-based quantitative proteomics analysis, we identified that NNMT was significantly increased in EGFR-TKI-resistant NSCLC cells. Moreover, we found that NNMT expression was increased in EGFR-TKI-resistant NSCLC tissue samples, and higher levels were correlated with shorter progression-free survival in EGFR-TKI-treated NSCLC patients. Knockdown of NNMT rendered EGFR-TKI-resistant cells more sensitive to EGFR-TKI, whereas overexpression of NNMT in EGFR-TKI-sensitive cells resulted in EGFR-TKI resistance. Mechanically, upregulation of NNMT increased c-myc expression via SIRT1-mediated c-myc deacetylation, which in turn promoted glycolysis and EGFR-TKI resistance. Furthermore, we demonstrated that the combination of NNMT inhibitor and EGFR-TKI strikingly suppressed the growth of EGFR-TKI-resistant NSCLC cells both in vitro and in vivo. In conclusion, our research indicated that NNMT overexpression is important for acquired resistance to EGFR-TKI and that targeting NNMT might be a potential therapeutic strategy to overcome resistance to EGFR TKI.

## Introduction

Lung cancer remains the leading cause of cancer-related mortality worldwide [1]. Non-small cell lung cancer (NSCLC) accounts for ~80% of lung cancer diagnoses. Activating mutations of epidermal growth factor receptor (EGFR) contributes to the progression of NSCLC and small-molecule inhibitors targeting EGFR (tyrosine kinase inhibitor, TKI), including the first- and second-generation EGFR-TKIs such as gefitinib, erlotinib, have demonstrated dramatic efficacy in NSCLC patients with EGFR-activating mutations [2]. Although EGFR-TKIs have a favorable and durable treatment response, most patients will eventually develop the progressive disease (PD) within about one year of treatment [3]. Different mechanisms of acquired resistance to EGFR-TKI, including EGFR T790M mutations, MET amplification, PIK3CA mutations, and phenotypic changes such as epithelial-mesenchymal transition (EMT), have been reported [4]. Recently, Osimertinib (AZD9291), a third-generation EGFR-TKI, showed a superior clinical response and outcome in EGFR-mutated NSCLC. However, the occurrence of acquired resistance to Osimertinib is inevitable, similar to that observed with other EGFR-TKIs. Further understanding of the underlying molecular mechanisms of EGFR-TKI resistance is still needed to reveal alternative strategies that can overcome EGFR-TKI resistance in patients.

Increased aerobic glycolysis has been recently discussed as a potential hallmark of cancer and is considered a possible therapeutic target for the treatment of cancers [5, 6]. Recent studies showed the pivotal regulation role of oncogenic EGFR on glycolysis in NSCLC, while treatment of NSCLC cells with EGFR-TKI reduces glucose-derived metabolites [7]. Suzuki et al. demonstrated an increase in GLUT1 expression and glucose uptake in EGFR-TKI resistant NSCLC cells and ablation of GLUT1 re-sensitizes resistant cells to gefitinib [8]. Treatment of EGFR-TKI resistant cells with the glucose analogue, 2-DG, improves the efficacy of the TKIs in EGFR mutated NSCLC models both in vitro and in vivo [9]. Moreover, metabolic properties of NSCLC can induce resistance to EGFR-TKI by reprograming stromal cells [10]. These studies suggested that EGFR mutations in NSCLC contribute to an increased reliance on glucose metabolism and EGFR-TKI resistance can be driven by enhanced glucose uptake.

Nicotinamide N-methyltransferase (NNMT), a S-adenosyl-l-methionine (SAM)-dependent methyltransferases that catalyzes the methylation of nicotinamide (NAM) to form 1-methylnicotinamide (MNAM), is an essential contributor to various metabolic and epigenetic processes [11]. Increased NNMT expression and activity have been implicated in several types of human cancers, including lung cancer [12, 13]. NNMT in malignant tumors plays a pivotal role in cell proliferation, migration, and metastasis [14, 15]; its overexpression is consequently correlated with poor prognosis in several cancers [16, 17]. Moreover, previous studies have demonstrated that overexpression of NNMT is associated with acquired resistance to chemotherapeutic agents or radiotherapy [18–22]. A recent investigation suggested an important role of NNMT in EGFR TKI resistance of EGFR-mutated NSCLC cells, and the addition of NNMT inhibitor could overcome this resistance [13]. However, the role and mechanism of NNMT in inducing acquired resistance to EGFR-TKI has not been fully investigated.

In this study, we uncovered a novel mechanism by which NNMT regulates EGFR-TKI resistance in NSCLC. We found that upregulation of NNMT increased c-myc expression via SIRT1-mediated c-myc deacetylation, which in turn promoted glycolysis and EGFR-TKI resistance. Moreover, we demonstrated that a combination of NNMT inhibitor and EGFR-TKI strikingly suppressed EGFR-TKI-resistant NSCLC cell growth both in vitro and in vivo, which might be a promising strategy for the treatment of NSCLC patients with acquired resistance to EGFR-TKI.

## Results

### Upregulation of NNMT in EGFR-TKI-resistant NSCLC cells and tissues

**Fig. 1:**
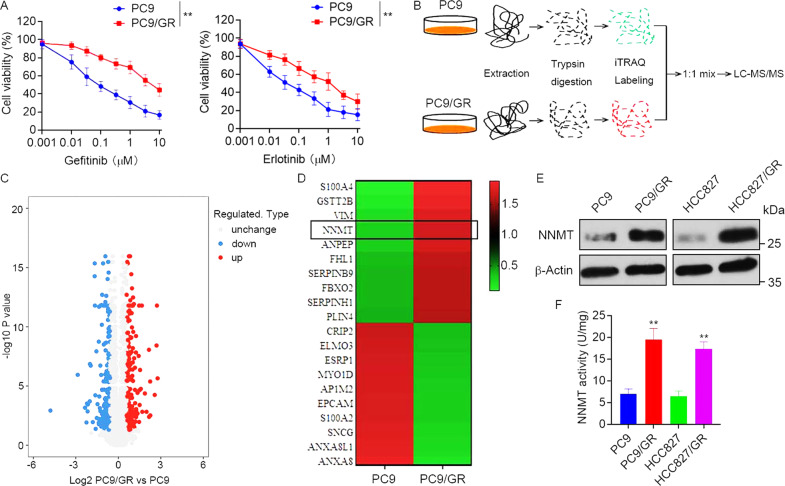
Upregulation of NNMT in EGFR-TKI-resistant NSCLC cells. **A** EGFR-TKI-resistant PC9/GR and parental PC9 cells were treated with gefitinib or erlotinib at the indicated concentration for 72 h, cell viability was evaluated by MTS assay. **B** iTRAQ-based quantitative proteomic comparison of PC9/GR cells and PC9 cells, experimental workflow showing the trypsin digestion, iTRAQ-labeling, and detection by tandem mass spectrometry. The acquired data was used to search a bioinformatics database. **C** Volcano plot showing the log_2_ fold-change and significance (−log_10_ *p*-value) of the proteome dataset. Red dots represent significantly upregulated proteins, blue dots represent significantly downregulated proteins. **D** Hierarchical clustering of top 20 differentially expressed proteins. **E** Validation of NNMT expression in EGFR-TKI-resistant PC9/GR, HCC827/GR, and parental PC9, HCC827 cells by Western blot. **F** NNMT specific activity in PC9/GR, HCC827/GR, PC9, and HCC827 cells was tested by measuring the amount of N^1^-methylnicotinamide produced. Each point represents the mean ± SD. Data show a representative of three independent experiments. (**p < 0.01).

To investigate the molecular mechanism of EGFR-TKI resistance in NSCLC, EGFR-TKI-resistant PC9/GR cells and the corresponding parental PC9 cells (Fig. 1A) were used for iTRAQ-based quantitative proteomics to identify differentially expressed proteins (Fig. 1B). A total of 5932 proteins were commonly quantified in both PC9/GR and PC9 cells. Among these, 533 proteins were significantly changed between PC9/GR and PC9 cells with thresholds of 1.5-fold changes and *p*-value ≤ 0.05 (Fig. 1C). Compared with EGFR-TKI sensitive PC9 cells, 267 proteins were upregulated, and 266 proteins were downregulated in PC9/GR cells (Supplementary Table 1). Owing to the proteins that could promote EGFR-TKI resistance, we focused on NNMT, which was one of the most upregulated proteins in PC9/GR with a 5.58-folds increase (Fig. 1D). We further validated the upregulation of NNMT in PC9/GR cells by Western blot (Fig. 1E). Moreover, the level of NNMT was dramatically higher in HCC827/GR cells, another EGFR-TKI-resistant NSCLC cell line, than that in parental HCC827 cells (Fig. S1, Fig. 1E). In keeping with the results of Western blot analysis, the level of NNMT specific activity was particularly increased in PC9/GR and HCC827/GR cells compared to PC9 and HCC827 cells, respectively (Fig. 1F).

**Fig. 2:**
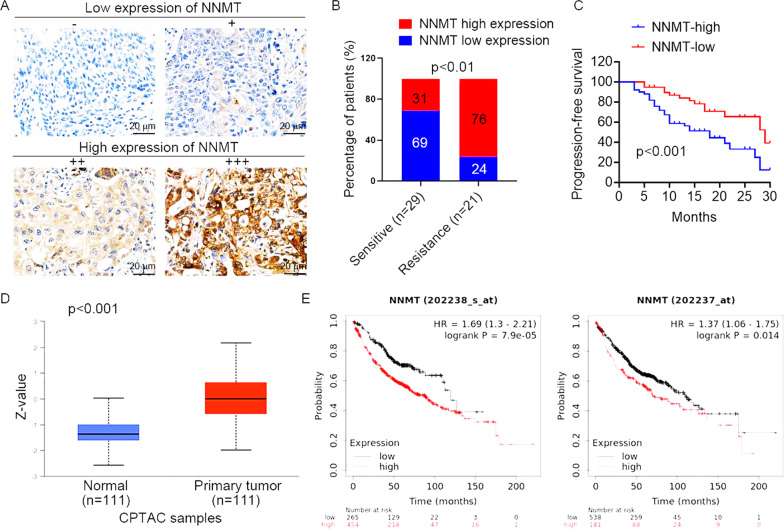
NNMT expression is associated with the response to EGFR-TKIs treatment in NSCLC. **A** Representative images of NNMT staining by immunohistochemistry analysis in NSCLC specimens. The intensity of NNMT staining was scored as 0 (no signal), 1 (weak), 2 (moderate), and 3 (marked). Percentage scores were assigned as 1, 1–25%; 2, 26–50%; 3, 51–75%; and 4, 76–100%. The scores of each tumor sample were multiplied to give a final score of 0–12, and the tumors were finally determined as negative (−), score 0; lower expression (+), score ≤4; moderate expression (++), score 5–8; and high expression (+++), score ≥9. **B** The percentages of patients with low expression (blue bar) and high expression of NNMT (red bar) were assigned according to different responses to EGFR-TKIs (gefitinib or erlotinib) (Sensitivity, *n* = 29; resistant, *n* = 21). **C** Survival curves of NSCLC patients with low expression versus high expression of NNMT taking EGFR-TKIs treatment. **D** NNMT expression in lung adenocarcinoma tissues and normal tissues based on the CPTAC database. **E** Kaplan–Meier OS curves (http://kmplot.com/analysis/) of NSCLC patients relative to different expression levels of NNMT (probe 202238_s_at, probe 202237_s_at).

We further determined by IHC detection whether NNMT was associated with the therapeutic effect of EGFR-TKI in NSCLC patients with EGFR mutations (*n* = 50) who received EGFR-TKI (gefitinib or erlotinib) treatment. Based on the divided groups of low NNMT expression (−/+) and high NNMT expression (++/+++) (Fig. 2A), an increased rate of high NNMT expression was observed in EGFR-TKI-resistant patients compared to EGFR-TKI-sensitive patients (Fig. 2B). Moreover, our collected cohort observed that upregulation of NNMT was associated with lower progression-free survival in NSCLC patients who received EGFR-TKI treatment (Fig. 2C). We further assessed the expression of NNMT by the Clinical Proteomic Tumor Analysis Consortium (CPTAC) database. The result showed that the protein expression of NNMT in lung adenocarcinoma was significantly higher than those in normal tissues (Fig. 2D), and the increase in NNMT protein expression level was positively correlated with cancer stage and tumor grade (Fig. S2). In addition, high levels of NNMT protein were significantly correlated with poor survival outcomes (Fig. 2E), according to an analysis of the online TCGA database. Collectively, these results suggested that patients with high expression of NNMT have a poor response to EGFR TKI, indicating that NNMT may be used to predict the efficacy of EGFR-TKI therapy in NSCLC patients.

### Effects of NNMT involved in EGFR-TKI resistance of NSCLC

**Fig. 3:**
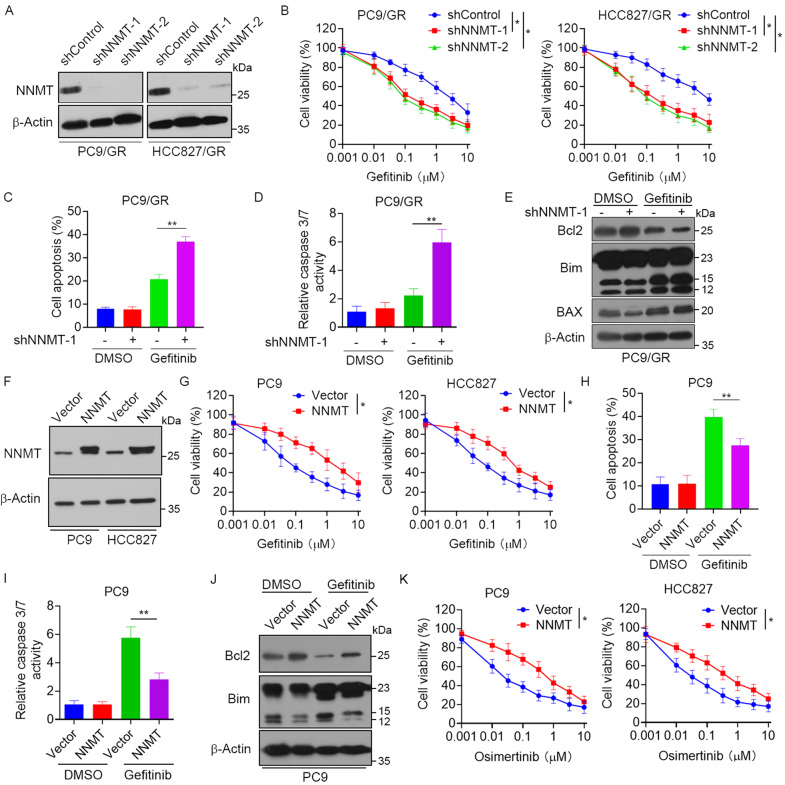
Effects of NNMT involved in EGFR-TKIs resistance of NSCLC. **A** PC9/GR or HCC827/GR cells were stably transfected with NNMT shRNA or control shRNA, the expression of NNMT and β-actin were measured by Western blot analysis. **B** PC9/GR or HCC827/GR cells transfected with NNMT shRNA or control shRNA were treated with gefitinib at the indicated concentration for 72 h, cell viability was evaluated by MTS assay. **C**–**E** PC9/GR cells transfected with NNMT shRNA or control shRNA were treated with 1 μM gefitinib for 48 h, **C** cells were stained with Annexin V-APC and propidium iodide, and the cell apoptosis was analyzed by flow cytometry, **D** Caspase 3/7 activity were measured, **E** the expression of Bcl2, Bim and BAX were measured by Western blot. **F** PC9 and HCC827 cells were stably transfected with NNMT-overexpressing vector, the expression of NNMT and β-actin were measured by Western blot. **G** PC9 and HCC827 cells transfected with NNMT-overexpressing vector were treated with gefitinib at the indicated concentration for 72 h, cell viability was evaluated by MTS assay. **H**–**J** PC9 cells transfected with NNMT-overexpressing vector were treated with 1 μM gefitinib for 48 h, **H** cells were stained with Annexin V-APC and propidium iodide, and the cell apoptosis were analyzed by flow cytometry, **I** Caspase 3/7 activity were measured, **J** the expression of Bcl2, Bim and BAX were measured by Western blot. **K** PC9 and HCC827 cells transfected with NNMT-overexpressing vector were treated with Osimertinib at the indicated concentration for 72 h, cell viability was evaluated by MTS assay. Each point represents the mean ± SD. Data show a representative of three independent experiments. (*p < 0.05, **p < 0.01).

To validate the role of NNMT in resistance EGFR-TKI resistance, we generated constructed stable NNMT-depleted PC9/GR and HCC827/GR cells (Fig. 3A) and found that knockdown of NNMT significantly increased sensitivity to gefitinib (Fig. 3B) and erlotinib (Fig. S3). Notably, knockdown of NNMT markedly increased gefitinib-induced cell apoptosis (Fig. 3C). Consistently, PC9/GR cells transfected with control shRNA showed a slight increase in caspase 3/7 activity after treatment of gefitinib. However, cells transfected with NNMT shRNA showed a significant increase in caspase 3/7 activity when incubated with gefitinib at the same concentrations (Fig. 3D). Moreover, knockdown of NNMT dramatically increased the expression of Bim and decreased the expression of Bcl-2 (Fig. 3E). In contrast, overexpression of NNMT in PC9 and HCC827 cells (Fig. 3F) exhibited increased resistance to gefitinib (Fig. 3G). Moreover, overexpression of NNMT significantly decreased gefitinib-induced cell apoptosis (Fig. 3H) and caspase3/7 activity (Fig. 3I). Furthermore, the treatment of gefitinib inhibited Bcl2 expression and promoted Bim expression. However, overexpression of NNMT dramatically reversed this effect (Fig. 3J). Notably, we also observed that overexpression of NNMT significantly increased resistance to the third-generation EGFR-TKI inhibitor Osimertinib (Fig. 3K). Collectively, these data suggested that elevated expression of NNMT was involved in the EGFR-TKI resistance of NSCLC.

### NNMT enhances glycolysis to promote EGFR-TKI resistance in NSCLC cells

**Fig. 4:**
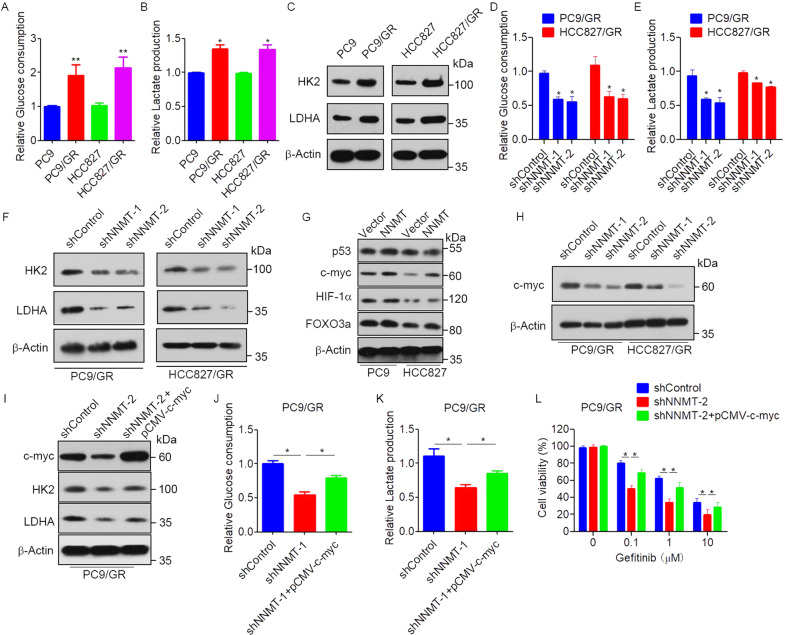
NNMT enhances glycolysis to promote EGFR-TKIs resistance in NSCLC cells. **A**, **B** Glucose uptake and Lactate secretion levels in PC9/GR, HCC827/GR, PC9, and HCC827 cells were measured. **C** The expression of HK2 and LDHA in PC9/GR, HCC827/GR, PC9, and HCC827 cells were measured by Western blot. **D**, **E** Glucose uptake and Lactate secretion levels in PC9/GR or HCC827/GR cells were stably transfected with NNMT shRNA or control shRNA. **F** PC9/GR or HCC827/GR cells were transfected with NNMT shRNA or control shRNA, the expression of HK2 and LDHA were measured by Western blot. **G** PC9 and HCC827 cells were stably transfected with NNMT-overexpressing vector, the expression of p53, c-myc, Hif-1α, FOXO3a, and β-actin were measured by Western blot. **H** PC9/GR or HCC827/GR cells were transfected with NNMT shRNA or control shRNA, the expression of HK2 and LDEA in were measured by Western blot. **I** PC9/GR cells were co-transfected with NNMT shRNA or control shRNA and pCMV-c-myc, the protein levels of c-myc, HK2, LDHA, and β-Actin were measured by western blot. **J**, **K** Glucose uptake and Lactate secretion levels in PC9/GR cells co-transfected with NNMT shRNA or control shRNA and pCMV-c-myc. **L** PC9/GR cells co-transfected with NNMT shRNA or control shRNA and pCMV-c-myc were treated with gefitinib at the indicated concentration for 72 h, cell viability was evaluated by MTS assay. Each point represents the mean ± SD. Data show a representative of three independent experiments. (*p < 0.05, **p < 0.01).

Previous studies suggested that glucose metabolism closely correlated with the drug resistance of cancer cells [23], and NNMT has emerged as a regulator of cancer energy metabolism [24]. Therefore, we hypothesized that NNMT mediated EGFR-TKI resistance via regulating of glucose metabolism in NSCLC cells. To test this hypothesis, the activity of glycolysis was examined in resistant and parental cells. The results showed that glucose consumption and lactate production were significantly elevated in EGFR-TKI-resistant PC9/GR and HCC827/GR cells compared with those in parental PC9 and HCC827 cells (Fig. 4A, B). Western blot results also showed that the expressions of glycolysis-related enzymes hexokinase 2 (HK2) and lactate dehydrogenase A (LDHA) were significantly increased in EGFR-TKI-resistant PC9/GR and HCC827/GR cells (Fig. 4C). Intriguingly, knockdown of NNMT in PC9/GR and HCC827/GR cells significantly decreased the glucose uptake, and lactate secretion (Fig. 4D, E). Moreover, knockdown of NNMT significantly decreased the expression of HK2, and LDHA (Fig. 4F). To identify potential factors that regulate glycolysis in response to NNMT-mediated EGFR-TKI resistance, we detected the regulation of NNMT on several transcription factors involved in glycolysis progression, including p53, c-myc, HIF-1α, FOXO3a. Western blot showed that overexpression of NNMT increased c-myc expression (Fig. 4G). However, overexpression of NNMT had no influence on p53, HIF-1α or FOXO3a (Fig. 4G). In contrast, the knockdown of NNMT significantly decreased c-myc expression (Fig. 4H). Moreover, overexpression of c-myc reversed the expression of HK2 and LDHA in NNMT-knockdown PC9/GR cells (Fig. 4I). Consistently, overexpression of c-myc dramatically increased glucose uptake and lactate secretion in NNMT-knockdown PC9/GR cells (Fig. 4J, K). Furthermore, overexpression of c-myc significantly decreased NNMT shRNA-induced gefitinib sensitivity in PC9/GR cells (Fig. 4L). These results indicated that NNMT promotes glycolysis and EGFR-TKI resistance in NSCLC cells via a c-myc-dependent mechanism.

### NNMT promotes glycolysis and EGFR-TKI resistance via a SIRT1-mediated c-myc deacetylation

**Fig. 5:**
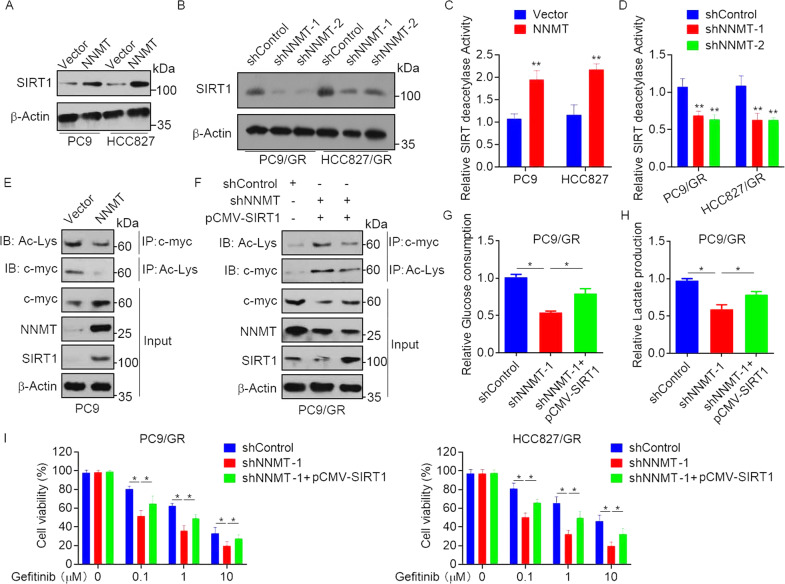
NNMT promotes glycolysis and EGFR-TKIs resistance via SIRT1-mediated c-myc deacetylation. **A**, **B** PC9 and HCC827 cells were stably transfected with NNMT-overexpressing vector (**A**), PC9/GR or HCC827/GR cells were transfected with NNMT shRNA or control shRNA (**B**), the expression of STAT1 and β-actin were measured by Western blot. **C**, **D** PC9 and HCC827 cells were stably transfected with NNMT-overexpressing vector (**C**), PC9/GR or HCC827/GR cells were transfected with NNMT shRNA or control shRNA (**D**), the SIRT1 activity levels were determined using a SIRT1 deacetylase fluorometric reagent kit. **E** Total protein extracts of PC9/GR cells that stably express NNMT or a control vector were subjected to IP using c-myc antibodies, followed by IB with Acetylated-Lysine Antibody and c-myc antibody. Reciprocal IP was done using Acetylated-Lysine Antibody, followed by IB with the c-myc antibody. **F** PC9/GR cells were co-transfected with NNMT shRNA or control shRNA and pCMV-SIRT1, the protein extracts were subjected to IP using c-myc antibodies, followed by IB with Acetylated-Lysine Antibody and c-myc antibody. Reciprocal IP was done using Acetylated-Lysine Antibody, followed by IB with the c-myc antibody. **G**, **H** Glucose uptake and Lactate secretion levels in PC9/GR cells co-transfected with NNMT shRNA or control shRNA and pCMV-SIRT1. **I** PC9/GR and HCC827/GR cells co-transfected with NNMT shRNA or control shRNA and pCMV-SIRT1 were treated with gefitinib at the indicated concentration for 72 h, cell viability was evaluated by MTS assay. Each point represents the mean ± SD. Data show a representative of three independent experiments. (*p < 0.05, **p < 0.01).

Previous study suggested that NNMT enhances chemoresistance through SIRT1 protein stabilization [18], and SIRT1-mediated deacetylation has been shown to play a critical role in the regulation of c-myc protein stability and expression [25]. We speculated that the enhancement of glycolysis and EGFR-TKI resistance by NNMT might be due to the upregulation of SIRT1. Supporting our assumption, we found that overexpression of NNMT increased SIRT1 protein expression in PC9 and HCC827 cells (Fig. 5A), whereas knockdown of NNMT significantly decreased SIRT1 protein expression in PC9/GR and HCC827/GR cells (Fig. 5B). Consistent with the SIRT1 protein level, SIRT1 deacetylation activity was significantly increased after NNMT overexpression and decreased after NNMT downregulation (Fig. 5C, D). We further examined whether the role of NNMT on c-myc expression is regulated by SIRT1-mediated deacetylation. The results showed that overexpression of NNMT decreased acetylation of c-myc (Fig. 5E). Moreover, we overexpressed SIRT1 in NNMT-knockdown PC9/GR cells and found that knockdown of NNMT resulted in hyperacetylation of c-myc, whereas overexpression of SIRT1 reversed NNMT shRNA-induced c-myc hyperacetylation and increased c-myc expression (Fig. 5F), suggesting that SIRT1-mediated deacetylation is involved in NNMT-induced c-myc expression. We next investigated the roles of SIRT1 in NNMT-induced EGFR-TKI resistance. The results showed that overexpression of SIRT1 dramatically increased glucose uptake and lactate secretion in NNMT-knockdown PC9/GR cells (Fig. 5G, H). Moreover, overexpression of SIRT1 significantly decreased NNMT shRNA-induced gefitinib sensitivity in PC9/GR and HCC827/GR cells (Fig. 5I). Together, these data indicated that NNMT promotes glycolysis and EGFR-TKI resistance dependents on SIRT1-mediated c-myc deacetylation.

### Pharmacological inhibition of NNMT overcomes EGFR-TKI resistance in NSCLC cells

**Fig. 6:**
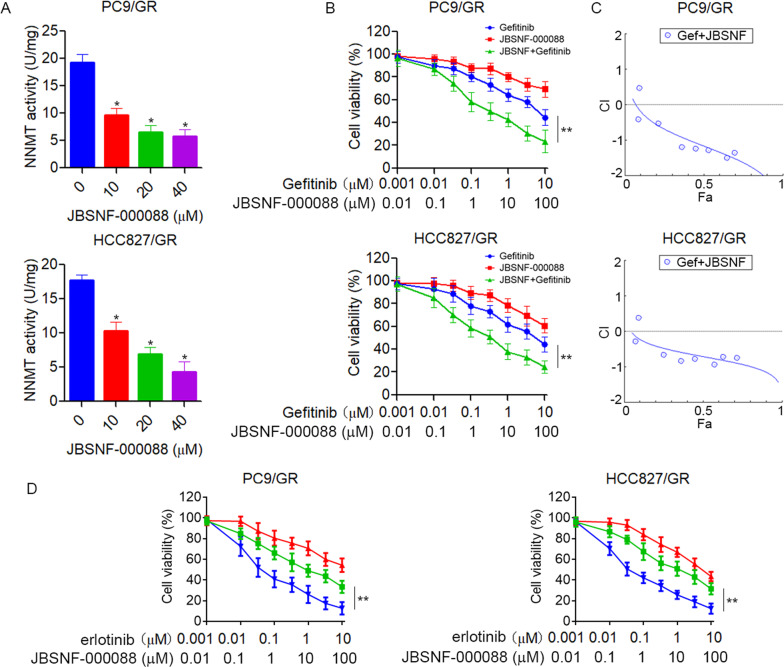
Pharmacological inhibition of NNMT overcomes EGFR-TKIs resistance in NSCLC cells in vitro. **A** PC9/GR or HCC827/GR cells were treated with NNMT inhibitor JBSNF-000088 at the indicated concentration for 48 h, NNMT specific activity was tested by measuring the amount of N^1^-methylnicotinamide produced. **B** PC9/GR or HCC827/GR cells were treated with gefitinib in combination with JBSNF-000088 at the indicated concentration for 72 h, cell viability was evaluated by MTS assay. **C** The combination index (CI) curves of gefitinib in combination with JBSNF-000088 were calculated using Calcusyn software according to the Chou–Talalay equation. **D** PC9/GR or HCC827/GR cells were treated with erlotinib in combination with JBSNF-000088 at the indicated concentration for 72 h, cell viability was evaluated by MTS assay. Each point represents the mean ± SD. Data show a representative of three independent experiments. (*p < 0.05, **p < 0.01).

**Fig. 7:**
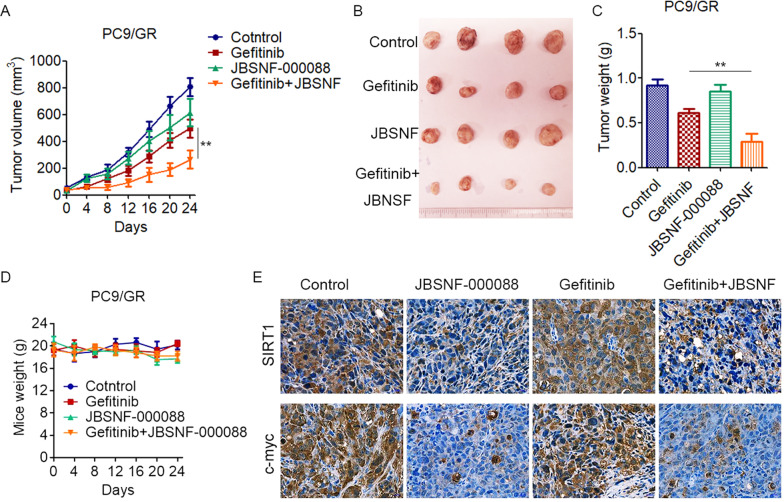
Pharmacological inhibition of NNMT overcomes EGFR-TKIs resistance in NSCLC cells in vivo. **A**–**C** 5 × 10^6^ PC9/GR cells were subcutaneously injected into nude mice and palpable tumors were allowed to develop for 7 days. Mice were randomly divided into four groups and treated with vehicle control (0.01% DMSO in PBS), gefitinib (50 mg/kg/d), JBSNF-000088 (50 mg/kg/d), or a combination of gefitinib with JBSNF-000088 every other day (*n* = 4, per group), **A** The tumor size was measured at indicated time intervals and calculated. The tumor volume was calculated using the formula: *V* = 1/2 × larger diameter × (smaller diameter)^2^, and growth curves were plotted using average tumor volume within each experimental group at the set time points, **B** At the end of treatment, tumors were excised, **C** Tumor weights were measured. **D** Treatment of gefitinib or/and JBSNF-000088 has no effect on mouse body weight. **E** PC9/GR xenograft tissues derived from mice were fixed, sectioned, and placed on slides. Tumor specimens were subjected to immunohistochemical staining with antibodies specific to SIRT and c-myc. (**p < 0.01).

Given that EGFR-TKI-resistant cells have increased NNMT and glycolysis, we explored whether pharmacological inhibition of NNMT could sensitize EGFR-TKI-resistant cells to EGFR-TKI. JBSNF-000088, a small molecule analog of NA, has been shown to inhibit NNMT activity [26]. Accordingly, treatment of JBSNF-000088 significantly inhibits NNMT activity in PC9/GR and HCC827/GR cells (Fig. 6A). Moreover, we found that the combination of JBSNF-000088 and gefitinib synergistically inhibit the growth of PC9/GR and HCC827/GR cells (Fig. 6B, C). Similar results were obtained with PC9/GR and HCC827/GR cells co-treated with JBSNF-000088 and erlotinib (Fig. 6D). To confirm these results in vivo, we tested the combination treatment of gefitinib and JBSNF-000088 in a xenograft nude mice model. PC9/GR cells were subcutaneously injected into 5-week-old nude mice. After tumors formed, the mice were randomly divided into four groups and treated tumor xenografts with gefitinib (50 mg/kg/days), JBSNF-000088 (50 mg/kg/days), or a combination of the agents. Consistent with our in vitro results, treatment with gefitinib, or JBSNF-000088 slightly reduced tumor growth; however, combined treatment with gefitinib plus JBSNF-000088 significantly inhibited tumor growth (Fig. 7A–C). All four groups of mice showed stable body weights, indicating that treatment with gefitinib or JBSNF-000088 was not associated with significant toxicity (Fig. 7D). Moreover, treatment of JBSNF-000088 significantly decreased the expression of SIRT1 and c-myc in xenograft tumor (Fig. 7E). These data further strengthened the hypotheses that pharmacological inhibition of NNMT overcoming EGFR-TKI resistance in NSCLC.

## Discussion

Although many patients harboring EGFR-activating mutations respond to EGFR-TKI treatment, the development of drug resistance remains the major therapeutic barrier in NSCLC [27]. Consequently, in depth understanding of the EGFR-TKI resistance mechanisms is of utmost importance for improving the therapeutic strategy. In this study, we showed that NNMT is crucial for EGFR-TKI resistance in NSCLC. Upregulation of NNMT increased c-myc expression via SIRT1-mediated c-myc deacetylation, which in turn promoted glycolysis and gefitinib resistance. Combined with NNMT inhibitor and gefitinib strikingly suppressed gefitinib-resistant NSCLC cells growth, which might be a promising strategy for the treatment of patients with acquired resistance to EGFR TKI.

Recent studies showed that NNMT is aberrantly expressed in several cancers and played an important role in cancer cell adhesion, invasion, and metastasis. A high level of NNMT is correlated with a poor prognosis for survival in cancer patients. Moreover, NNMT overexpression has recently been found to be associated with chemotherapy resistance [18, 19, 21]. Using proteomics analysis, we observed that NNMT were significantly increased in EGFR-TKI-resistant NSCLC cells which is consistent with the previous observation that the basal levels of NNMT expression in gefitinib-resistant NSCLC cells were overexpressed compared with their parental NSCLC cells [13]. Notably, we found a high level of NNMT expression correlated with poor survival and EGFR-TKI response in NSCLC patients. Moreover, we demonstrated that knockdown of NNMT reversed gefitinib or erlotinib resistance in NSCLC cells. Similar to NNMT knockdown, the combination of NNMT inhibitor and EGFR-TKI treatment significantly overcame EGFR-TKI resistance and showed a significant synergistic effect in inhibition of EGFR-TKI resistant NSCLC cells. These data suggest that targeting NNMT can be a novel strategy to overcome EGFR-TKI resistance in lung cancer.

Cancer cells predominantly utilize glycolysis to sustain their characteristic uncontrolled growth and proliferation [28]. Recent studies have emphasized the importance role of glycolysis therapeutic resistance [29, 30]. Notably, increasing evidences have demonstrated that glycolysis is elevated in EGFR-mutated NSCLC cells with acquired EGFR-TKI resistance [8], and targeting glycolytic enzymes has been shown to improve therapeutic response to EGFR TKI in NSCLC [9, 31, 32]. Considering the impact of glycolysis on EGFR-TKI resistance, we confirmed that NNMT is sufficient to induce EGFR-TKI resistance in conjunction with elevated glycolysis in this study. We found that knockdown of NNMT significantly reduced glucose consumption and lactate production in EGFR-TKI-resistant NSCLC cells, whereas overexpression of NNMT increased glucose consumption and lactate production. Moreover, Western blot analysis results showed that NNMT promoted the protein expression of glycolysis-related enzymes HK2 and LDHA. Consistent with our studies, Cui et al. revealed that NNMT knockdown significantly increased the sensitivity of xenografts to 5-FU and suppressed the Warburg effect in esophageal squamous cell carcinoma [21].

NNMT conversion of NAM to MNAM effectively diverts NAM from being recycled back to nicotinamide adenine dinucleotide (NAD+) by the NAM salvage pathway and affects global NAD+ levels. Thus, NNMT appears to be a critical metabolic enzyme linking NAD+ metabolism to the control of gene expression via its ability to regulate NAD+ levels [24, 33]. Previous studies suggested that NAD+ is required for the activity of SIRT1, and higher NAD+ levels can promote the deacetylase activity of SIRT1 [34]. Notably, we found that knockdown of NNMT significantly decreased SIRT1 expression and activity, whereas overexpression of NNMT increased SIRT1 expression and activity. Moreover, we found that overexpression of SIRT1 significantly decreased NNMT shRNA-induced gefitinib sensitivity in EGFR-TKI-resistant NSCLC cells, suggesting that SIRT1 is involved in NNMT-mediated gefitinib resistance in NSCLC cells. SIRT1 is a key regulator of cellular metabolism via the deacetylation of target proteins [35, 36]. Previous studies have revealed a critical role for SIRT1-mediated deacetylation in the regulation of c-myc protein stability [25]. c-myc has been known to regulate glycolysis at multiple steps of glucose metabolism [37]. c-myc increases glucose uptake through directly transactivation of glucose transporter 1 [38]. c-myc activation also increases glycolysis through the activation of HK2 and LDHA [39, 40]. Here, we demonstrated that NNMT could promote glycolysis in a SIRT1-dependent c-myc deacetylation. Ectopic expression of NNMT decreased acetylation of c-myc, followed by upregulation of c-myc and downstream glycolytic genes of LDHA, and HK2, which led to increased glycolytic activity.

In summary, we uncovered a novel oncogenic signaling pathway involved in NNMT that exclusively acts in acquired EGFR-TKI resistance by activating SIRT1/c-myc signaling and promoting glycolysis. Moreover, we suggested that targeting NNMT could be a potential therapeutic strategy to enhance the efficacy of EGFR TKI in patients with acquired resistance.

## Materials and methods

### Cells and culture condition

Human NSCLC cell lines (PC9, HCC827) were from the American Type Culture Collection (ATCC, Manassas, VA, USA). The gefitinib-resistant NSCLC cell model (PC9/GR, HCC827/GR) was successfully established by continually exposing PC9 and HCC827 cells to a gradually increasing concentration of gefitinib for 6 months. Gefitinib (Sigma-Aldrich, St. Louis, MO, USA) was added to the culture medium at a concentration of 0.1 μmol/L to sustain the resistance phenotype of PC9/GR and HCC827/GR cells. All cells were cultured in RPMI 1640 medium supplemented with 10% FBS in a 37 °C humidified atmosphere containing 95% air and 5% CO_2_. Cells were authenticated with DNA profiling by short tandem repeat (STR) analysis.

### RNA interference and plasmid transfection

Lentiviral vectors expressing nontargeting pLKO.1 control shRNA (SCH002), and two shRNA constructs targeting NNMT-shRNA1 (TRCN0000035224) and -shRNA2 (TRCN0000035225) were obtained from Sigma-Aldrich. Expression plasmid for pCMV6-NNMT, pCMV6-SIRT1, pCMV6-c-myc, and pCMV6-XL5 empty plasmid were purchased from Origene (Rockville, MD). For cell transfection, Lipofectamine 2000 reagent (Invitrogen) was used according to the manufacturer’s instructions. After 72 h infection, stably expressing or knockdown cells were selected in RPMI 1640 medium containing 1 μg/mL puromycin.

### Cell viability assay

Cell viability was determined using The CellTiter 96 AQueous One Solution Cell Proliferation Assay (Promega, Madison, WI, USA). Briefly, cells were plated onto 96-well plates with a medium containing 10% FBS. After 12 h, the culture medium was replaced with a fresh medium containing 5% FBS (control), or the same medium containing different concentrations of gefitinib or JBSNF-000088 (MedChemExpress LLC, Shanghai, China). After 72 h, the MTS reagent was added to the cell culture. Cells were then incubated at 37 °C for an additional 1 h, and the absorbance was measured by a Synergy LX Multi-Mode Reader (Biotek, Winooski, VT, USA).

### Cell apoptosis assay

Cell apoptosis was determined by Annexin V-APC/PI apoptosis kit (Keygen Biotech, Nanjing, China), followed by flow cytometer analysis (BD FACSCalibur flow cytometer, Becton & Dickinson Company, Franklin Lakes, NJ, USA) according to the manufacturer’s instructions. The percentages of apoptotic cells in the Annexin V+/PI− and Annexin V+/PI+ populations were determined.

### Western blot

Cells were lysed in RIPA buffer (50 mM Tris-Cl, pH 8.0, 150 mM NaCl, 5 mM EDTA, 0.1% SDS, 1% NP-40) supplemented with protease inhibitor cocktail. Cell lysates were centrifuged at 12,000 rpm for 30 min at 4 °C, supernatants were saved, and protein concentrations were determined by BCA protein assay (Thermo Scientific, Rockford, Illinois, USA). Equal amounts of total cell lysates or CM of cell culture were subjected to western blot assays as we described previously [41, 42] to measure protein expression. Antibodies for western blot analyses were from the following sources: NNMT rabbit mAb (E6N2Z, #33361), SIRT1 mouse mAb (1F3, #8469), Hexokinase II rabbit mAb (C64G5, #2867), LDHA rabbit mAb (C4B5, #3582), c-myc rabbit mAb (E5Q6W, #18583), BAX Rabbit mAb (D2E11, #5023), Bim Rabbit mAb (C34C5, #2933), Bcl2 Rabbit mAb (D55G8 #4223), FOXO3a rabbit mAb (D19A7, #12829), p53 Mouse mAb (1C12, #2524), Acetylated-Lysine Antibody (#9441), and β-actin mouse mAb (8H10D10, #3700) (Cell Signaling Technology, Beverly, MA, USA).

### Immunoprecipitation

Cells were lysed in a buffer containing 2 mmol/L Tris-HCl (pH7.4), 10 mmol/L EDTA, 100 mmol/L NaCl and 1% IGEPAL. Cell lysates containing 500 μg of total protein were incubated with specific antibodies (2 μg) for 18 h at 4 °C with constant rotation. After that, 50 μl of protein G agarose beads were added to incubate for 3 h. The beads were washed five times with lysis buffer. The precipitated proteins were detected by Western blot.

### NNMT enzyme assay

An HPLC-based catalytic assay was performed to analyze NNMT activity as described [43]. Briefly, 5 × 10^6^ cells were suspended in 200 µl of lysis buffer (50 mM tris-HCl, pH 8.6, 2 µg/ml aprotinin, 1 mM phenylmethylsulfonyl fluoride, 1 mM dithiothreitol, 1% Nonidet P40) and ½ volume of glass beads. The homogenate was centrifuged at 16,000 × *g* for 10 min at 4 °C. The supernatant was collected at 4 °C until assayed. The standard assay mixture contained 50 mM tris-HCl, pH 8.6, 1 mM dithiothreitol, 5 mM nicotinamide, 0.5 mM SAM and the appropriate amount of enzyme sample to a reach final volume of 350 µl. The reaction was started by adding the substrate SAM. Incubations were performed at 37 °C for 30 and 60 min. The reaction was stopped by adding 100 µl assay mixture to 50 µl ice-cold 1.2 M HClO_4_. Proteins were removed by 1 min of centrifugation in a microfuge and 130 µl perchloric acid supernatant were then neutralized by adding 35 µl 0.8 M K_2_CO_3_. 100 µl of the neutralized supernatant was injected into a high-performance liquid chromatography system 10 Dvp-uv-vis photodiode array detector using a 250 × 4.6 mm inner diameter Supelcosil® LC-18 5 µm reversed-phase column. Enzyme activities were tested by measuring the amount of N^1^-methylnicotinamide produced, as determined by the peak areas of the separated compound with 1 U activity representing the formation of 1 nmol N^1^- methylnicotinamide per hour of incubation at 37 °C.

### SIRT1 activity assay

The intracellular SIRT1 activities were measured using a SIRT1 deacetylase fluorometric reagent kit (SIRT1 Deacetylase Fluorometric Assay Kit, CycLex Co., Ltd., Japan). Briefly, 1 × 10^7^ cells were harvested and resuspended in 1 mL of lysate buffer. The supernatant was discarded after 13,000 × *g* for 10 min at 4 °C. After the protein concentration was determined using the BCA protein assay (Thermo Scientific, Rockford, Illinois, USA), the activity of SIRT1 in the nuclear protein fraction was measured according to the manufacturer’s instructions. Each experiment was conducted at least three times.

### Caspase-3/7 activity assay

Caspase activity was measured by Caspase-Glo 3/7 Assay kit (Promega, Madison, WI, USA) according to the manufacturer’s protocol.

### Glucose consumption and lactate production assay

Cells (2 × 10^5^/well) were cultured in 6-well plates at 37 °C for 48 h. The glucose content in the medium was detected using a Glucose Assay kit (cat. no. GAGO20; Sigma-Aldrich; Merck KGaA), and the lactate content in the medium was detected using a Lactate Assay kit (cat. no. MAK064; Sigma-Aldrich; Merck KGaA), all according to the manufacturer’s protocols. Samples were analyzed using a Synergy LX Multi-Mode Reader (Biotek, Winooski, VT, USA). The rates of glucose consumption and lactate production were calculated according to the standard curve line and OD value of each sample.

### Immunohistochemistry

Tumor tissues from 50 cases of EGFR mutant lung adenocarcinoma patients who had received EGFR-TKIs (erlotinib or gefitinib) treatment after surgery were investigated; these patients were enrolled from December 2012 to January 2016. Response to treatment was evaluated according to the Response Evaluation Criteria in Solid Tumors. This study was approved by the Ethics Committee of Guangzhou Medical University.

Immunohistochemistry assays were performed as we described previously [44]. The intensity of NNMT staining was scored as 0 (no signal), 1 (weak), 2 (moderate), and 3 (marked). Percentage scores were assigned as 1, 1–25%; 2, 26–50%; 3, 51–75%; and 4, 76–100%. The scores of each tumor sample were multiplied to give a final score of 0–12, and the tumors were finally determined as negative (−), score 0; lower expression (+), score ≤4; moderate expression (++), score 5–8; and high expression (+++), score ≥9. An optimal cutoff value was identified: a staining index of five or greater was used to define tumors of high expression, and four or lower for low expression.

### Animal experiments

Five-week-old female Balb/c-nude mice were purchased from the Guangdong Animal Experimental Center. 5 × 10^5^ gefitinib-resistant PC9/GR cells were subcutaneously injected into nude mice. When the average tumor volume reached 50 mm^3^, mice were randomly divided into four groups (*n* = 4, per group) and treated with PBS, gefitinib (50 mg kg^−1^ per days, i.g.), JBSNF-000088 (50 mg kg^−1^ per days, i.g.), or combination of gefitinib and JBSNF-000088 every other day. The volume of the tumor was calculated following the formula: Volume = Length × Width^2^ × 0.5. At the end of this experiment, tumors were harvested and weighed, and then were dissected for fixing and embedding in paraffin. The animal studies were approved by the Institutional Animal Care and Use Committee (IACUC) of Guangzhou Medical University. Standard animal care and laboratory guidelines were conducted according to the IACUC protocol.

iTRAQ-based proteomics assay is described in the supplemental experimental procedure.

### Statistical analysis

Data were analyzed using SPSS 22.0 statistical software. All experiments were repeated at least thrice. The experimental data are expressed as the mean ± standard deviation (SD). Students *t*-test was used to compare the means of two groups of independent samples, and results with *P* < 0.05 were considered statistically significant.

## Supplementary information

Supplemental information

Unprocessed western blots

## Data Availability

All data generated or analyzed during this study are included in this published article (as well as in the accompanying Supporting Information).
