# Supplementary material for: Targeting nicotinamide N-methyltransferase overcomes resistance to EGFR-TKI in non-small cell lung cancer cells
Source: Cell Death Discov. 2022 Apr 6;8:170. doi: 10.1038/s41420-022-00966-x (PMC8986855; doi:10.1038/s41420-022-00966-x)
Supplement: Supplementary file 1 — Supplemental information [file 41420_2022_966_MOESM1_ESM.docx]

**Supplemental information**

**Targeting Nicotinamide N-methyltransferase overcomes resistance to EGFR-TKI in Non-Small Cell Lung Cancer Cells**

Jun Wang, Xi Liu, Yuanfeng Huang, Pan Li, Minqiang Yang, Shanshan Zeng, Danyang Chen, Qian Wang, Hao Liu, Kai Luo, Jin Deng

**Supplementary Figures**

**Figures S1**

**
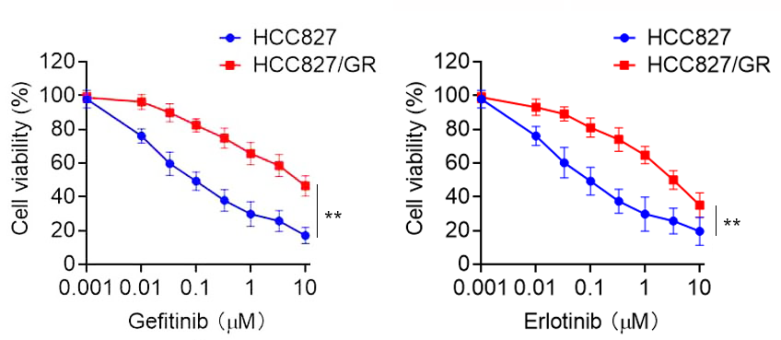
**

**Figure. S1.** EGFR-TKIs-resistant HCC827/GR and parental HCC827 cells were treated with gefitinib or erlotinib at indicated concentration for 72 h, cell viability was evaluated by MTS assay. (** p<0.01)

**Figures S2**


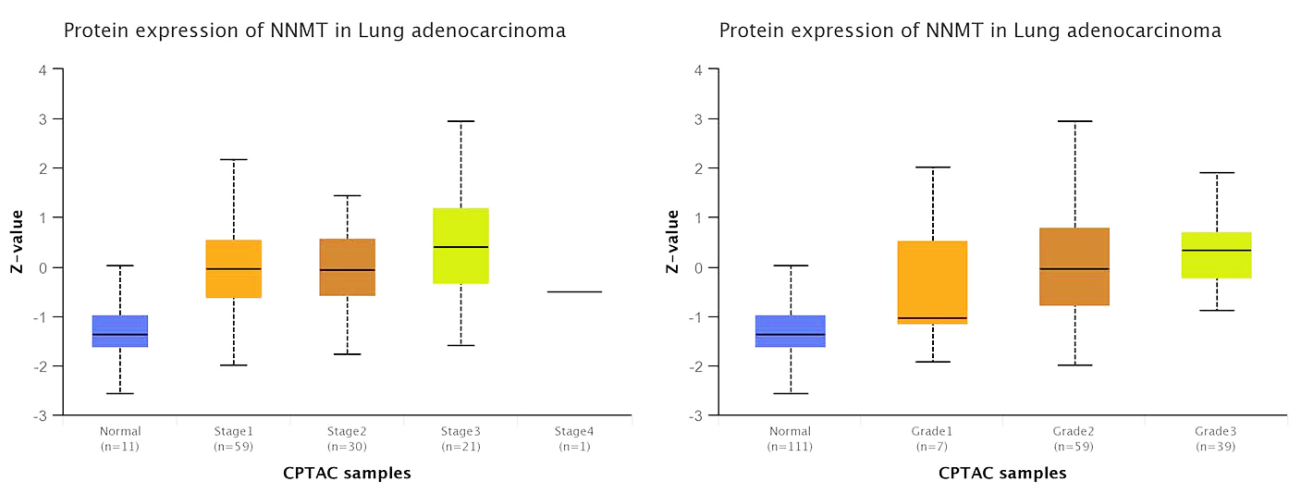


**Figure. S2** NNMT expression in lung adenocarcinoma tissues and normal tissues base on CPTAC database.

**Figures S3**


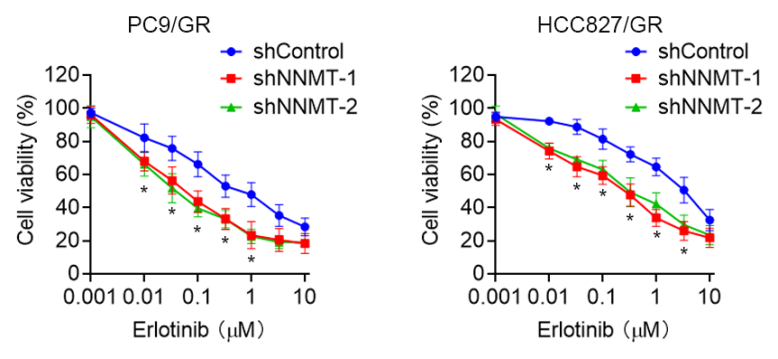


**Figure. S3** PC9/GR or HCC827/GR cells transfected with NNMT shRNA or control shRNA were treated with erlotinib at indicated concentration for 72 h, cell viability was evaluated by MTS assay. (* p<0.05)

**Supplemental Experimental Procedures**

**iTRAQ-based proteomics analysis**

**Protein Extraction**

Cells were sonicated three times on ice using a high intensity ultrasonic processor (Scientz) in lysis buffer (8 M urea, 1% Protease Inhibitor Cocktail). The remaining debris was removed by centrifugation at 12,000 g at 4 °C for 10 min. Finally, the supernatant was collected and the protein concentration was determined with BCA kit according to the manufacturer’s instructions.

**Trypsin Digestion**

For digestion, the protein solution was reduced with 5 mM dithiothreitol for 30 min at 56 °C and alkylated with 11 mM iodoacetamide for 15 min at room temperature in darkness. The protein sample was then diluted by adding 100 mM TEAB to urea concentration less than 2M. Finally, trypsin was added at 1:50 trypsin-to-protein mass ratio for the first digestion overnight and 1:100 trypsin-to-protein mass ratio for a second 4 h-digestion.

**TMT/iTRAQ Labeling**

After trypsin digestion, peptide was desalted by Strata X C18 SPE column (Phenomenex) and vacuum-dried. Peptide was reconstituted in 0.5 M TEAB and processed according to the manufacturer’s protocol for TMT kit/iTRAQ kit. Briefly, one unit of TMT/iTRAQ reagent were thawed and reconstituted in acetonitrile. The peptide mixtures were then incubated for 2 h at room temperature and pooled, desalted and dried by vacuum centrifugation.

**LC-MS/MS Analysis**

The tryptic peptides were dissolved in 0.1% formic acid (solvent A), directly loaded onto a home-made reversed-phase analytical column (15-cm length, 75 μm i.d.). The gradient was comprised of an increase from 6% to 23% solvent B (0.1% formic acid in 98% acetonitrile) over 26 min, 23% to 35% in 8 min and climbing to 80% in 3 min then holding at 80% for the last 3 min, all at a constant flow rate of 400 nL/min on an EASY-nLC 1000 UPLC system.

The peptides were subjected to NSI source followed by tandem mass spectrometry (MS/MS) in Q ExactiveTM Plus (Thermo) coupled online to the UPLC. The electrospray voltage applied was 2.0 kV. The m/z scan range was 350 to 1800 for full scan, and intact peptides were detected in the Orbitrap at a resolution of 70,000. Peptides were then selected for MS/MS using NCE setting as 28 and the fragments were detected in the Orbitrap at a resolution of 17,500. A data-dependent procedure that alternated between one MS scan followed by 20 MS/MS scans with 15.0s dynamic exclusion. Automatic gain control (AGC) was set at 5E4. Fixed first mass was set as 100 m/z.

**Database Search**

The resulting MS/MS data were processed using Maxquant search engine (v.1.5.2.8). Tandem mass spectra were searched against human uniprot database concatenated with reverse decoy database. Trypsin/P was specified as cleavage enzyme allowing up to 4 missing cleavages. The mass tolerance for precursor ions was set as 20 ppm in First search and 5 ppm in Main search, and the mass tolerance for fragment ions was set as 0.02 Da. Carbamidomethyl on Cys was specified as fixed modification and Acetylation modification and oxidation on Met were specified as variable modifications. FDR was adjusted to < 1% and minimum score for modified peptides was set > 40.

**Supplementary Table**

**Table S1.** Differentially expressed proteins between gefitinib-resistant PC9/GR cells and the corresponding parental PC9 cells

| **Protein accession** | **Protein description** | **PC9GR/PC9 Ratio** | **Regulated Type** | **PC9GR/PC9 P value** | **Gene name** | **MW [kDa]** | **Score** | **Coverage [%]** | **Peptides** | **PSMs** | **Unique peptides** | **PC9** | **PC9/GR** |
| --- | --- | --- | --- | --- | --- | --- | --- | --- | --- | --- | --- | --- | --- |
| P26447 | Protein S100-A4 | 19.8 | Up | 0.0015 | S100A4 | 11.728 | 8.6397 | 25.7 | 4 | 9 | 4 | 0.095 | 1.881 |
| P0CG30 | Glutathione S-transferase theta-2B | 12.099 | Up | 0.0124 | GSTT2B | 27.507 | 19.598 | 21.3 | 4 | 7 | 4 | 0.151 | 1.827 |
| P08670 | Vimentin | 7.576 | Up | 1.62E-12 | VIM | 53.651 | 323.31 | 67.8 | 36 | 176 | 33 | 0.229 | 1.735 |
| P40261 | Nicotinamide N-methyltransferase | 5.585 | Up | 4.03E-06 | NNMT | 29.574 | 30.233 | 36.4 | 8 | 14 | 8 | 0.301 | 1.681 |
| P15144 | Aminopeptidase N | 5.498 | Up | 1.64E-12 | ANPEP | 109.54 | 134.79 | 25.3 | 22 | 45 | 22 | 0.305 | 1.677 |
| Q13642 | Four and a half LIM domains protein 1 | 4.263 | Up | 0.0055 | FHL1 | 36.263 | 18.499 | 13.3 | 4 | 5 | 4 | 0.377 | 1.607 |
| P50453 | Serpin B9 | 4.048 | Up | 0.000425 | SERPINB9 | 42.403 | 47.946 | 25.3 | 8 | 12 | 7 | 0.393 | 1.591 |
| Q9UK22 | F-box only protein 2 | 4.038 | Up | 0.0198 | FBXO2 | 33.327 | 57.75 | 17.2 | 4 | 7 | 4 | 0.394 | 1.591 |
| P50454 | Serpin H1 | 3.987 | Up | 1.62E-12 | SERPINH1 | 46.44 | 323.31 | 51.4 | 20 | 69 | 20 | 0.398 | 1.587 |
| Q96Q06 | Perilipin-4 | 3.841 | Up | 0.0034 | PLIN4 | 134.43 | 135.78 | 32.1 | 16 | 21 | 16 | 0.41 | 1.575 |
| P26038 | Moesin | 3.627 | Up | 1.62E-12 | MSN | 67.819 | 294.79 | 51.3 | 36 | 118 | 25 | 0.429 | 1.556 |
| Q01995 | Transgelin | 3.463 | Up | 0.000375 | TAGLN | 22.611 | 44.532 | 37.3 | 7 | 11 | 7 | 0.445 | 1.541 |
| Q32P28 | Prolyl 3-hydroxylase 1 | 3.461 | Up | 1.77E-05 | P3H1 | 83.393 | 41.701 | 15.6 | 9 | 14 | 9 | 0.445 | 1.54 |
| P27487 | Dipeptidyl peptidase 4 | 3.365 | Up | 0.0454 | DPP4 | 88.278 | 11.407 | 7.7 | 6 | 8 | 6 | 0.455 | 1.531 |
| Q14315 | Filamin-C | 3.292 | Up | 0.0433 | FLNC | 291.02 | 10.098 | 5.8 | 16 | 34 | 4 | 0.463 | 1.524 |
| P08133 | Annexin A6 | 3.208 | Up | 1.55E-06 | ANXA6 | 75.872 | 60.233 | 33.3 | 20 | 24 | 20 | 0.472 | 1.514 |
| Q15847 | Adipogenesis regulatory factor | 3.181 | Up | 5.92E-05 | ADIRF | 7.8547 | 24.168 | 27.6 | 2 | 6 | 2 | 0.475 | 1.511 |
| O43491 | Band 4.1-like protein 2 | 2.974 | Up | 1.62E-12 | EPB41L2 | 112.59 | 259.57 | 32.4 | 28 | 46 | 26 | 0.5 | 1.487 |
| Q16555 | Dihydropyrimidinase-related protein 2 | 2.937 | Up | 1.62E-12 | DPYSL2 | 62.293 | 197.26 | 49.8 | 20 | 48 | 17 | 0.505 | 1.483 |
| Q02809 | Procollagen-lysine,2-oxoglutarate 5-dioxygenase 1 | 2.896 | Up | 2.61E-12 | PLOD1 | 83.549 | 146.36 | 31.9 | 19 | 35 | 19 | 0.51 | 1.477 |
| Q9HAV0 | Guanine nucleotide-binding protein subunit beta-4 | 2.86 | Up | 0.000239 | GNB4 | 37.567 | 57.816 | 24.4 | 8 | 21 | 4 | 0.515 | 1.473 |
| P22676 | Calretinin | 2.758 | Up | 1.11E-16 | CALB2 | 31.54 | 131.33 | 52.4 | 15 | 29 | 15 | 0.529 | 1.459 |
| P37235 | Hippocalcin-like protein 1 | 2.744 | Up | 3E-11 | HPCAL1 | 22.313 | 178.36 | 47.2 | 9 | 22 | 9 | 0.531 | 1.457 |
| Q8NBJ5 | Procollagen galactosyltransferase 1 | 2.569 | Up | 1.67E-12 | COLGALT1 | 71.635 | 86.262 | 29.7 | 17 | 38 | 17 | 0.557 | 1.431 |
| Q9Y5V3 | Melanoma-associated antigen D1 | 2.563 | Up | 0.00338 | MAGED1 | 86.16 | 25.226 | 4.6 | 3 | 4 | 2 | 0.558 | 1.43 |
| Q14914 | Prostaglandin reductase 1 | 2.412 | Up | 4.86E-09 | PTGR1 | 35.869 | 48.657 | 30.1 | 9 | 13 | 9 | 0.583 | 1.406 |
| P17252 | Protein kinase C alpha type | 2.406 | Up | 1.18E-07 | PRKCA | 76.749 | 31.181 | 14.3 | 8 | 12 | 8 | 0.584 | 1.405 |
| Q5SSJ5 | Heterochromatin protein 1-binding protein 3 | 2.406 | Up | 2.28E-05 | HP1BP3 | 61.206 | 39.131 | 21.2 | 12 | 14 | 12 | 0.584 | 1.405 |
| P21266 | Glutathione S-transferase Mu 3 | 2.402 | Up | 0.00108 | GSTM3 | 26.559 | 36.27 | 30.7 | 6 | 9 | 6 | 0.585 | 1.405 |
| Q63ZY3 | KN motif and ankyrin repeat domain-containing protein 2 | 2.394 | Up | 1.98E-05 | KANK2 | 91.173 | 15.932 | 7.3 | 5 | 6 | 5 | 0.586 | 1.403 |
| P13987 | CD59 glycoprotein | 2.356 | Up | 1.65E-05 | CD59 | 14.177 | 31.396 | 25.8 | 5 | 15 | 5 | 0.593 | 1.397 |
| O00592 | Podocalyxin | 2.317 | Up | 0.0158 | PODXL | 58.635 | 27.464 | 5.2 | 3 | 5 | 3 | 0.6 | 1.39 |
| Q9UM22 | Mammalian ependymin-related protein 1 | 2.317 | Up | 0.0393 | EPDR1 | 25.437 | 13.541 | 9.4 | 2 | 3 | 2 | 0.6 | 1.39 |
| P80723 | Brain acid soluble protein 1 | 2.282 | Up | 1.52E-13 | BASP1 | 22.693 | 104.47 | 48.5 | 7 | 18 | 7 | 0.606 | 1.383 |
| O94992 | Protein HEXIM1 | 2.278 | Up | 0.0477 | HEXIM1 | 40.623 | 32.667 | 13.6 | 3 | 4 | 3 | 0.607 | 1.383 |
| Q13445 | Transmembrane emp24 domain-containing protein 1 | 2.268 | Up | 1.88E-10 | TMED1 | 25.206 | 23.101 | 20.3 | 5 | 8 | 5 | 0.609 | 1.381 |
| Q86UT6 | NLR family member X1 | 2.268 | Up | 0.0492 | NLRX1 | 107.61 | 16.011 | 7.6 | 7 | 8 | 7 | 0.609 | 1.381 |
| O75718 | Cartilage-associated protein | 2.252 | Up | 2.68E-07 | CRTAP | 46.561 | 40.33 | 23.4 | 9 | 14 | 9 | 0.612 | 1.378 |
| Q8IVF2 | Protein AHNAK2 | 2.246 | Up | 1E-32 | AHNAK2 | 616.62 | 323.31 | 29 | 64 | 105 | 63 | 0.613 | 1.377 |
| Q6UW68 | Transmembrane protein 205 | 2.241 | Up | 1.55E-07 | TMEM205 | 21.198 | 20.917 | 27 | 4 | 9 | 4 | 0.614 | 1.376 |
| Q9Y2G5 | GDP-fucose protein O-fucosyltransferase 2 | 2.241 | Up | 0.00138 | POFUT2 | 49.975 | 13.713 | 17 | 7 | 9 | 7 | 0.614 | 1.376 |
| O95833 | Chloride intracellular channel protein 3 | 2.22 | Up | 0.0262 | CLIC3 | 26.648 | 19.823 | 26.3 | 5 | 5 | 5 | 0.618 | 1.372 |
| P26885 | Peptidyl-prolyl cis-trans isomerase FKBP2 | 2.21 | Up | 0.000157 | FKBP2 | 15.649 | 26.271 | 28.9 | 4 | 7 | 4 | 0.62 | 1.37 |
| P42330 | Aldo-keto reductase family 1 member C3 | 2.205 | Up | 0.0435 | AKR1C3 | 36.853 | 10.589 | 11.5 | 3 | 4 | 3 | 0.621 | 1.369 |
| P50914 | 60S ribosomal protein L14 | 2.184 | Up | 1.14E-07 | RPL14 | 23.432 | 34.178 | 19.1 | 4 | 12 | 4 | 0.625 | 1.365 |
| P16401 | Histone H1.5 | 2.179 | Up | 0.000443 | HIST1H1B | 22.58 | 29.774 | 21.7 | 6 | 14 | 4 | 0.626 | 1.364 |
| P20020 | Plasma membrane calcium-transporting ATPase 1 | 2.174 | Up | 0.00152 | ATP2B1 | 138.75 | 76.474 | 17.6 | 16 | 27 | 7 | 0.627 | 1.363 |
| O75923 | Dysferlin | 2.165 | Up | 0.00752 | DYSF | 237.29 | 6.8925 | 2.6 | 5 | 7 | 4 | 0.629 | 1.362 |
| Q9Y696 | Chloride intracellular channel protein 4 | 2.165 | Up | 1.48E-07 | CLIC4 | 28.772 | 79.694 | 37.5 | 9 | 21 | 9 | 0.629 | 1.362 |
| Q14938 | Nuclear factor 1 X-type | 2.16 | Up | 0.0166 | NFIX | 55.098 | 4.4039 | 4.8 | 2 | 3 | 1 | 0.63 | 1.361 |
| Q9NUJ3 | T-complex protein 11-like protein 1 | 2.155 | Up | 0.000622 | TCP11L1 | 57.034 | 13.979 | 4.3 | 2 | 3 | 2 | 0.631 | 1.36 |
| Q9UHD9 | Ubiquilin-2 | 2.139 | Up | 0.00178 | UBQLN2 | 65.695 | 73.311 | 25.8 | 10 | 18 | 5 | 0.634 | 1.356 |
| Q93045 | Stathmin-2 | 2.139 | Up | 0.0175 | STMN2 | 20.828 | 11.028 | 10.6 | 2 | 6 | 1 | 0.634 | 1.356 |
| Q6NYC8 | Phostensin | 2.139 | Up | 0.000223 | PPP1R18 | 67.942 | 32.649 | 15 | 7 | 8 | 7 | 0.634 | 1.356 |
| P02751 | Fibronectin | 2.139 | Up | 4.51E-08 | FN1 | 262.62 | 50.79 | 7.9 | 14 | 19 | 14 | 0.634 | 1.356 |
| O60888 | Protein CutA | 2.135 | Up | 9.75E-05 | CUTA | 19.116 | 18.028 | 15.6 | 3 | 7 | 3 | 0.635 | 1.356 |
| Q9H074 | Polyadenylate-binding protein-interacting protein 1 | 2.134 | Up | 0.0043 | PAIP1 | 53.524 | 26.411 | 15 | 6 | 6 | 6 | 0.635 | 1.355 |
| P16070 | CD44 antigen | 2.131 | Up | 3.48E-08 | CD44 | 81.537 | 28.588 | 9.6 | 8 | 24 | 8 | 0.636 | 1.355 |
| P09913 | Interferon-induced protein with tetratricopeptide repeats 2 | 2.126 | Up | 2.57E-10 | IFIT2 | 54.632 | 66.81 | 21 | 9 | 14 | 9 | 0.637 | 1.354 |
| P01024 | Complement C3 | 2.126 | Up | 0.00826 | C3 | 187.15 | 29.922 | 5.7 | 8 | 8 | 8 | 0.637 | 1.354 |
| P43121 | Cell surface glycoprotein MUC18 | 2.124 | Up | 9.8E-07 | MCAM | 71.607 | 35.789 | 11.8 | 7 | 10 | 7 | 0.637 | 1.353 |
| P46821 | Microtubule-associated protein 1B | 2.121 | Up | 2.07E-10 | MAP1B | 270.63 | 91.688 | 10.1 | 19 | 22 | 19 | 0.638 | 1.353 |
| P60033 | CD81 antigen | 2.119 | Up | 0.000085 | CD81 | 25.809 | 19.96 | 15.3 | 2 | 6 | 2 | 0.638 | 1.352 |
| P05556 | Integrin beta-1 | 2.116 | Up | 1.62E-12 | ITGB1 | 88.414 | 156.81 | 23.6 | 18 | 47 | 18 | 0.639 | 1.352 |
| P17693 | HLA class I histocompatibility antigen, alpha chain G | 2.111 | Up | 0.0113 | HLA-G | 38.224 | 8.8314 | 9.5 | 3 | 8 | 1 | 0.64 | 1.351 |
| Q9NZW5 | MAGUK p55 subfamily member 6 | 2.109 | Up | 0.000362 | MPP6 | 61.116 | 29.176 | 13.1 | 7 | 9 | 6 | 0.64 | 1.35 |
| Q14195 | Dihydropyrimidinase-related protein 3 | 2.106 | Up | 0.0277 | DPYSL3 | 61.963 | 40.442 | 24.6 | 12 | 17 | 9 | 0.641 | 1.35 |
| P21291 | Cysteine and glycine-rich protein 1 | 2.106 | Up | 1.98E-09 | CSRP1 | 20.567 | 89.265 | 46.6 | 7 | 20 | 7 | 0.641 | 1.35 |
| P21980 | Protein-glutamine gamma-glutamyltransferase 2 | 2.082 | Up | 1.62E-12 | TGM2 | 77.328 | 87.875 | 25.5 | 16 | 38 | 16 | 0.646 | 1.345 |
| Q9ULC5 | Long-chain-fatty-acid--CoA ligase 5 | 2.082 | Up | 0.0022 | ACSL5 | 75.99 | 38.977 | 16.7 | 8 | 11 | 7 | 0.646 | 1.345 |
| Q01628 | Interferon-induced transmembrane protein 3 | 2.073 | Up | 0.00186 | IFITM3 | 14.632 | 31.609 | 18.8 | 1 | 3 | 1 | 0.648 | 1.343 |
| P32119 | Peroxiredoxin-2 | 2.068 | Up | 1.62E-12 | PRDX2 | 21.892 | 20.729 | 39.9 | 8 | 27 | 7 | 0.649 | 1.342 |
| O95865 | N(G),N(G)-dimethylarginine dimethylaminohydrolase 2 | 2.063 | Up | 0.00294 | DDAH2 | 29.644 | 14.989 | 25.6 | 7 | 11 | 6 | 0.65 | 1.341 |
| Q96CX2 | BTB/POZ domain-containing protein KCTD12 | 2.058 | Up | 1.66E-05 | KCTD12 | 35.7 | 33.598 | 19.1 | 5 | 10 | 5 | 0.651 | 1.34 |
| P61769 | Beta-2-microglobulin | 2.054 | Up | 3.55E-05 | B2M | 13.714 | 8.2378 | 16.8 | 2 | 6 | 2 | 0.652 | 1.339 |
| O94808 | Glutamine--fructose-6-phosphate aminotransferase [isomerizing] 2 | 2.049 | Up | 3.95E-09 | GFPT2 | 76.93 | 38.981 | 27.4 | 15 | 19 | 12 | 0.653 | 1.338 |
| P23921 | Ribonucleoside-diphosphate reductase large subunit | 2.044 | Up | 2.41E-10 | RRM1 | 90.069 | 71.676 | 23.7 | 16 | 29 | 16 | 0.654 | 1.337 |
| Q09666 | Neuroblast differentiation-associated protein AHNAK | 2.044 | Up | 1E-32 | AHNAK | 629.09 | 323.31 | 63.3 | 233 | 669 | 232 | 0.654 | 1.337 |
| P09382 | Galectin-1 | 2.044 | Up | 1.62E-12 | LGALS1 | 14.716 | 142.3 | 58.5 | 8 | 61 | 8 | 0.654 | 1.337 |
| Q6NZI2 | Caveolae-associated protein 1 | 2.04 | Up | 1.62E-12 | CAVIN1 | 43.476 | 184.15 | 35.6 | 10 | 27 | 10 | 0.655 | 1.336 |
| P16949 | Stathmin | 2.021 | Up | 1.12E-08 | STMN1 | 17.302 | 25.43 | 35.6 | 7 | 20 | 6 | 0.659 | 1.332 |
| A6NDU8 | UPF0600 protein C5orf51 | 2.017 | Up | 0.01 | C5orf51 | 33.62 | 23.267 | 14.6 | 2 | 3 | 2 | 0.66 | 1.331 |
| P29508 | Serpin B3 | 2.003 | Up | 5.52E-05 | SERPINB3 | 44.564 | 11.302 | 14.4 | 6 | 8 | 6 | 0.663 | 1.328 |
| O95816 | BAG family molecular chaperone regulator 2 | 2.003 | Up | 7.56E-09 | BAG2 | 23.772 | 69.625 | 36.5 | 8 | 11 | 8 | 0.663 | 1.328 |
| Q70UQ0 | Inhibitor of nuclear factor kappa-B kinase-interacting protein | 2 | Up | 0.000156 | IKBIP | 39.309 | 20.645 | 33.4 | 13 | 15 | 13 | 0.664 | 1.328 |
| Q96FZ2 | Embryonic stem cell-specific 5-hydroxymethylcytosine-binding protein | 0.496 | Down | 0.00552 | HMCES | 40.574 | 22.659 | 18.1 | 5 | 6 | 5 | 1.343 | 0.666 |
| Q9BX68 | Histidine triad nucleotide-binding protein 2, mitochondrial | 0.495 | Down | 1.84E-05 | HINT2 | 17.162 | 57.108 | 27.6 | 3 | 5 | 3 | 1.344 | 0.665 |
| P34897 | Serine hydroxymethyltransferase, mitochondrial | 0.49 | Down | 1.62E-12 | SHMT2 | 55.992 | 280.59 | 50.8 | 21 | 51 | 20 | 1.348 | 0.661 |
| Q8TBX8 | Phosphatidylinositol 5-phosphate 4-kinase type-2 gamma | 0.489 | Down | 4.68E-08 | PIP4K2C | 47.299 | 45.758 | 15 | 8 | 12 | 8 | 1.349 | 0.66 |
| Q99986 | Serine/threonine-protein kinase VRK1 | 0.486 | Down | 1.42E-09 | VRK1 | 45.476 | 30.775 | 30.1 | 10 | 13 | 10 | 1.352 | 0.657 |
| P13498 | Cytochrome b-245 light chain | 0.484 | Down | 0.000236 | CYBA | 21.012 | 11.626 | 12.8 | 2 | 7 | 2 | 1.354 | 0.655 |
| Q27J81 | Inverted formin-2 | 0.483 | Down | 1.62E-12 | INF2 | 135.62 | 266.85 | 24.3 | 22 | 36 | 22 | 1.355 | 0.654 |
| P35221 | Catenin alpha-1 | 0.479 | Down | 1.62E-12 | CTNNA1 | 100.07 | 323.31 | 43.4 | 31 | 62 | 31 | 1.358 | 0.651 |
| Q9Y6D6 | Brefeldin A-inhibited guanine nucleotide-exchange protein 1 | 0.477 | Down | 4.96E-12 | ARFGEF1 | 208.76 | 98.478 | 16.2 | 27 | 36 | 17 | 1.361 | 0.649 |
| Q92552 | 28S ribosomal protein S27, mitochondrial | 0.477 | Down | 0.00166 | MRPS27 | 47.611 | 15.27 | 12.1 | 5 | 7 | 5 | 1.36 | 0.649 |
| Q12774 | Rho guanine nucleotide exchange factor 5 | 0.475 | Down | 2.19E-06 | ARHGEF5 | 176.8 | 8.2978 | 3.3 | 4 | 4 | 4 | 1.362 | 0.647 |
| P55061 | Bax inhibitor 1 | 0.474 | Down | 0.00506 | TMBIM6 | 26.537 | 1.7778 | 3.4 | 1 | 2 | 1 | 1.363 | 0.646 |
| Q92599 | Septin-8 | 0.473 | Down | 0.0398 | SEPT8 | 55.756 | 13.856 | 19 | 7 | 11 | 4 | 1.364 | 0.645 |
| P49327 | Fatty acid synthase | 0.473 | Down | 1.62E-12 | FASN | 273.42 | 323.31 | 43.4 | 85 | 240 | 85 | 1.364 | 0.645 |
| Q9NVI1 | Fanconi anemia group I protein | 0.471 | Down | 0.00238 | FANCI | 149.32 | 21.811 | 7.2 | 8 | 9 | 8 | 1.366 | 0.643 |
| Q8NFV4 | Protein ABHD11 | 0.47 | Down | 4.36E-05 | ABHD11 | 34.69 | 48.103 | 30.8 | 7 | 11 | 7 | 1.367 | 0.643 |
| O43278 | Kunitz-type protease inhibitor 1 | 0.469 | Down | 0.000744 | SPINT1 | 58.397 | 11.662 | 9.8 | 5 | 7 | 5 | 1.368 | 0.641 |
| P33316 | Deoxyuridine 5'-triphosphate nucleotidohydrolase, mitochondrial | 0.467 | Down | 5.42E-08 | DUT | 26.563 | 43.575 | 38.9 | 9 | 20 | 9 | 1.369 | 0.64 |
| Q53H82 | Endoribonuclease LACTB2 | 0.466 | Down | 1.62E-12 | LACTB2 | 32.805 | 88.549 | 43.4 | 10 | 23 | 10 | 1.371 | 0.639 |
| Q9P1F3 | Costars family protein ABRACL | 0.466 | Down | 0.001 | ABRACL | 9.0564 | 10.689 | 16 | 1 | 3 | 1 | 1.371 | 0.639 |
| Q99575 | Ribonucleases P/MRP protein subunit POP1 | 0.464 | Down | 2.44E-15 | POP1 | 114.71 | 116.48 | 27.2 | 23 | 39 | 23 | 1.373 | 0.637 |
| P04183 | Thymidine kinase, cytosolic | 0.462 | Down | 8.86E-07 | TK1 | 25.468 | 28.648 | 39.3 | 8 | 11 | 8 | 1.375 | 0.635 |
| P28676 | Grancalcin | 0.462 | Down | 1.29E-07 | GCA | 24.01 | 11.096 | 18.9 | 5 | 7 | 5 | 1.375 | 0.635 |
| P0DN79 | Cystathionine beta-synthase-like protein | 0.461 | Down | 0.0453 | CBSL | 60.586 | 25.273 | 6.5 | 3 | 4 | 3 | 1.375 | 0.634 |
| Q9UHR4 | Brain-specific angiogenesis inhibitor 1-associated protein 2-like protein 1 | 0.46 | Down | 2.55E-09 | BAIAP2L1 | 56.882 | 104.09 | 31.3 | 14 | 21 | 14 | 1.376 | 0.633 |
| Q9GZR7 | ATP-dependent RNA helicase DDX24 | 0.459 | Down | 5.31E-11 | DDX24 | 96.331 | 76.956 | 20.4 | 17 | 26 | 17 | 1.377 | 0.632 |
| P08195 | 4F2 cell-surface antigen heavy chain | 0.459 | Down | 1.62E-12 | SLC3A2 | 67.993 | 306.85 | 33.8 | 19 | 46 | 19 | 1.378 | 0.632 |
| Q96AB3 | Isochorismatase domain-containing protein 2 | 0.458 | Down | 0.0003 | ISOC2 | 22.337 | 72.641 | 34.6 | 5 | 8 | 5 | 1.379 | 0.631 |
| Q13740 | CD166 antigen | 0.454 | Down | 5.55E-16 | ALCAM | 65.102 | 80.357 | 27.6 | 15 | 29 | 15 | 1.382 | 0.628 |
| Q9UBP9 | PTB domain-containing engulfment adapter protein 1 | 0.453 | Down | 0.000103 | GULP1 | 34.49 | 10.671 | 6.6 | 2 | 3 | 2 | 1.383 | 0.626 |
| P02144 | Myoglobin | 0.45 | Down | 1.53E-05 | MB | 17.184 | 17.089 | 44.8 | 5 | 7 | 5 | 1.386 | 0.624 |
| Q96NY8 | Nectin-4 | 0.448 | Down | 0.0121 | NECTIN4 | 55.454 | 7.8502 | 5.1 | 2 | 3 | 2 | 1.388 | 0.622 |
| Q12955 | Ankyrin-3 | 0.443 | Down | 0.000601 | ANK3 | 480.4 | 9.1329 | 1 | 4 | 4 | 4 | 1.393 | 0.617 |
| P11413 | Glucose-6-phosphate 1-dehydrogenase | 0.44 | Down | 1E-32 | G6PD | 59.256 | 263.46 | 57.5 | 29 | 84 | 29 | 1.396 | 0.614 |
| Q8NC26 | Zinc finger protein 114 | 0.439 | Down | 0.00208 | ZNF114 | 47.746 | 15.077 | 13.9 | 5 | 7 | 5 | 1.397 | 0.613 |
| P68366 | Tubulin alpha-4A chain | 0.438 | Down | 5.35E-07 | TUBA4A | 49.924 | 31.665 | 58.3 | 21 | 142 | 4 | 1.398 | 0.612 |
| P55327 | Tumor protein D52 | 0.436 | Down | 3.4E-10 | TPD52 | 24.327 | 130.94 | 32.1 | 5 | 12 | 5 | 1.4 | 0.61 |
| Q8TDB6 | E3 ubiquitin-protein ligase DTX3L | 0.436 | Down | 7.03E-10 | DTX3L | 83.553 | 132.18 | 19.9 | 13 | 15 | 13 | 1.4 | 0.61 |
| Q8WWI1 | LIM domain only protein 7 | 0.428 | Down | 0.00316 | LMO7 | 192.69 | 14.345 | 3.3 | 5 | 6 | 5 | 1.408 | 0.603 |
| P29373 | Cellular retinoic acid-binding protein 2 | 0.428 | Down | 1.52E-08 | CRABP2 | 15.693 | 49.03 | 29 | 4 | 7 | 4 | 1.408 | 0.602 |
| P04424 | Argininosuccinate lyase | 0.425 | Down | 6.32E-11 | ASL | 51.657 | 58.149 | 23.3 | 10 | 17 | 10 | 1.411 | 0.6 |
| Q14451 | Growth factor receptor-bound protein 7 | 0.417 | Down | 0.0208 | GRB7 | 59.68 | 25.539 | 14.5 | 6 | 7 | 6 | 1.419 | 0.592 |
| P08236 | Beta-glucuronidase | 0.416 | Down | 0.000145 | GUSB | 74.731 | 7.6777 | 3.8 | 3 | 5 | 3 | 1.419 | 0.591 |
| Q03169 | Tumor necrosis factor alpha-induced protein 2 | 0.414 | Down | 8.51E-13 | TNFAIP2 | 72.66 | 184.11 | 26.3 | 16 | 27 | 16 | 1.422 | 0.589 |
| Q8IXK2 | Polypeptide N-acetylgalactosaminyltransferase 12 | 0.414 | Down | 0.00562 | GALNT12 | 66.938 | 33.267 | 13.9 | 6 | 7 | 5 | 1.422 | 0.589 |
| A6NFQ2 | TRPM8 channel-associated factor 2 | 0.413 | Down | 1.72E-05 | TCAF2 | 100.9 | 23.637 | 10 | 8 | 9 | 8 | 1.423 | 0.587 |
| Q14435 | Polypeptide N-acetylgalactosaminyltransferase 3 | 0.412 | Down | 0.0407 | GALNT3 | 72.609 | 9.2022 | 7.9 | 4 | 4 | 4 | 1.424 | 0.587 |
| Q9H4G0 | Band 4.1-like protein 1 | 0.408 | Down | 0.000504 | EPB41L1 | 98.502 | 32.91 | 13.8 | 11 | 12 | 10 | 1.428 | 0.583 |
| P47895 | Aldehyde dehydrogenase family 1 member A3 | 0.408 | Down | 1.63E-06 | ALDH1A3 | 56.108 | 16.779 | 14.3 | 7 | 8 | 5 | 1.428 | 0.583 |
| P09601 | Heme oxygenase 1 | 0.407 | Down | 0.0046 | HMOX1 | 32.818 | 48.512 | 24 | 5 | 7 | 5 | 1.429 | 0.582 |
| P04792 | Heat shock protein beta-1 | 0.406 | Down | 1E-32 | HSPB1 | 22.782 | 197.46 | 81 | 14 | 70 | 14 | 1.43 | 0.581 |
| Q14192 | Four and a half LIM domains protein 2 | 0.406 | Down | 0.0013 | FHL2 | 32.193 | 20.072 | 21.5 | 6 | 6 | 6 | 1.43 | 0.581 |
| Q8IXQ6 | Poly [ADP-ribose] polymerase 9 | 0.398 | Down | 9.1E-08 | PARP9 | 96.342 | 46.664 | 15.9 | 14 | 15 | 14 | 1.438 | 0.573 |
| Q9UEW8 | STE20/SPS1-related proline-alanine-rich protein kinase | 0.393 | Down | 0.00016 | STK39 | 59.473 | 58.814 | 17.1 | 6 | 11 | 5 | 1.444 | 0.568 |
| Q9H6S3 | Epidermal growth factor receptor kinase substrate 8-like protein 2 | 0.393 | Down | 1.69E-05 | EPS8L2 | 80.62 | 33.8 | 11.3 | 8 | 10 | 8 | 1.444 | 0.568 |
| P35222 | Catenin beta-1 | 0.393 | Down | 2.34E-14 | CTNNB1 | 85.496 | 303.25 | 33.2 | 21 | 43 | 18 | 1.444 | 0.567 |
| P20591 | Interferon-induced GTP-binding protein Mx1 | 0.391 | Down | 0.00208 | MX1 | 75.519 | 28.123 | 12.4 | 8 | 11 | 8 | 1.446 | 0.566 |
| P08243 | Asparagine synthetase [glutamine-hydrolyzing] | 0.391 | Down | 1.44E-15 | ASNS | 64.369 | 74.839 | 32.6 | 16 | 37 | 16 | 1.446 | 0.566 |
| Q13268 | Dehydrogenase/reductase SDR family member 2, mitochondrial | 0.388 | Down | 1.62E-12 | DHRS2 | 29.926 | 66.748 | 41.8 | 9 | 33 | 9 | 1.449 | 0.562 |
| P14923 | Junction plakoglobin | 0.374 | Down | 1.58E-13 | JUP | 81.744 | 198.21 | 36.1 | 20 | 33 | 17 | 1.464 | 0.548 |
| P31641 | Sodium- and chloride-dependent taurine transporter | 0.37 | Down | 4.03E-07 | SLC6A6 | 69.829 | 5.9262 | 4.2 | 2 | 5 | 2 | 1.469 | 0.543 |
| O14896 | Interferon regulatory factor 6 | 0.362 | Down | 0.0141 | IRF6 | 53.129 | 60.666 | 17.1 | 8 | 12 | 8 | 1.477 | 0.535 |
| P07476 | Involucrin | 0.36 | Down | 0.00184 | IVL | 68.478 | 35.517 | 18.6 | 7 | 8 | 7 | 1.479 | 0.533 |
| O43175 | D-3-phosphoglycerate dehydrogenase | 0.356 | Down | 2.86E-11 | PHGDH | 56.65 | 102.89 | 30.6 | 14 | 26 | 14 | 1.485 | 0.528 |
| Q6ZT62 | Bargin | 0.349 | Down | 0.0132 | BARGIN | 73.599 | 15.188 | 8 | 5 | 7 | 4 | 1.492 | 0.521 |
| Q9Y446 | Plakophilin-3 | 0.346 | Down | 6.03E-13 | PKP3 | 87.081 | 67.669 | 23.1 | 16 | 27 | 16 | 1.495 | 0.518 |
| P52566 | Rho GDP-dissociation inhibitor 2 | 0.343 | Down | 0.000104 | ARHGDIB | 22.988 | 19.717 | 13.4 | 2 | 3 | 2 | 1.498 | 0.514 |
| P21964 | Catechol O-methyltransferase | 0.342 | Down | 1.62E-12 | COMT | 30.037 | 185.89 | 48 | 11 | 36 | 11 | 1.5 | 0.513 |
| Q6DT37 | Serine/threonine-protein kinase MRCK gamma | 0.34 | Down | 0.000621 | CDC42BPG | 172.46 | 20.779 | 5.2 | 7 | 7 | 6 | 1.502 | 0.511 |
| O15231 | Zinc finger protein 185 | 0.336 | Down | 9.07E-08 | ZNF185 | 73.525 | 82.005 | 32.7 | 17 | 22 | 17 | 1.507 | 0.506 |
| Q9NQU5 | Serine/threonine-protein kinase PAK 6 | 0.322 | Down | 4.28E-05 | PAK6 | 74.868 | 46.935 | 17.8 | 8 | 10 | 8 | 1.523 | 0.49 |
| Q9Y2T7 | Y-box-binding protein 2 | 0.321 | Down | 0.0115 | YBX2 | 38.517 | 13.548 | 22.5 | 6 | 13 | 3 | 1.502 | 0.482 |
| Q8NFU3 | Thiosulfate:glutathione sulfurtransferase | 0.315 | Down | 0.00038 | TSTD1 | 12.53 | 7.3228 | 26.1 | 3 | 5 | 3 | 1.532 | 0.482 |
| P23743 | Diacylglycerol kinase alpha | 0.304 | Down | 3.02E-07 | DGKA | 82.629 | 12.513 | 4.4 | 3 | 3 | 3 | 1.544 | 0.47 |
| Q13938 | Calcyphosin | 0.295 | Down | 9.76E-05 | CAPS | 20.967 | 31.925 | 25.4 | 5 | 10 | 5 | 1.555 | 0.459 |
| P01011 | Alpha-1-antichymotrypsin | 0.274 | Down | 0.00138 | SERPINA3 | 47.65 | 6.6568 | 2.6 | 1 | 2 | 1 | 1.582 | 0.433 |
| P12830 | Cadherin-1 | 0.269 | Down | 5.78E-05 | CDH1 | 97.455 | 64.319 | 13 | 9 | 15 | 8 | 1.589 | 0.427 |
| Q9P2M7 | Cingulin | 0.254 | Down | 0.000004 | CGN | 136.38 | 61.01 | 10.7 | 11 | 12 | 11 | 1.607 | 0.408 |
| O00515 | Ladinin-1 | 0.248 | Down | 0.00062 | LAD1 | 57.13 | 59.28 | 17.2 | 7 | 12 | 7 | 1.574 | 0.391 |
| P25815 | Protein S100-P | 0.246 | Down | 1.91E-05 | S100P | 10.4 | 5.3419 | 10.5 | 1 | 4 | 1 | 1.618 | 0.398 |
| Q8NI99 | Angiopoietin-related protein 6 | 0.243 | Down | 0.0124 | ANGPTL6 | 51.694 | 1.8548 | 2.8 | 1 | 2 | 1 | 1.622 | 0.394 |
| P09758 | Tumor-associated calcium signal transducer 2 | 0.238 | Down | 2.02E-05 | TACSTD2 | 35.709 | 16.177 | 16.4 | 5 | 11 | 5 | 1.629 | 0.387 |
| P36952 | Serpin B5 | 0.233 | Down | 1.04E-09 | SERPINB5 | 42.1 | 135.36 | 41.1 | 12 | 20 | 12 | 1.635 | 0.381 |
| P52943 | Cysteine-rich protein 2 | 0.225 | Down | 0.000943 | CRIP2 | 22.492 | 28.476 | 28.8 | 4 | 7 | 4 | 1.647 | 0.37 |
| Q96BJ8 | Engulfment and cell motility protein 3 | 0.215 | Down | 1.53E-06 | ELMO3 | 81.466 | 23.087 | 11.2 | 8 | 9 | 8 | 1.66 | 0.357 |
| Q6NXG1 | Epithelial splicing regulatory protein 1 | 0.203 | Down | 0.0179 | ESRP1 | 75.585 | 84.676 | 17.9 | 9 | 12 | 8 | 1.676 | 0.341 |
| O94832 | Unconventional myosin-Id | 0.193 | Down | 0.00818 | MYO1D | 116.2 | 36.563 | 9.7 | 8 | 10 | 8 | 1.692 | 0.326 |
| Q9Y6Q5 | AP-1 complex subunit mu-2 | 0.187 | Down | 0.0007 | AP1M2 | 48.108 | 10.241 | 11.6 | 5 | 6 | 3 | 1.7 | 0.318 |
| P16422 | Epithelial cell adhesion molecule | 0.181 | Down | 1.79E-06 | EPCAM | 34.932 | 85.906 | 31.5 | 7 | 16 | 7 | 1.709 | 0.309 |
| P29034 | Protein S100-A2 | 0.179 | Down | 7.84E-05 | S100A2 | 11.117 | 3.9722 | 17.3 | 2 | 5 | 2 | 1.712 | 0.306 |
| O76070 | Gamma-synuclein | 0.157 | Down | 0.0323 | SNCG | 13.331 | 42.889 | 25.2 | 3 | 4 | 3 | 1.745 | 0.274 |
| Q5VT79 | Annexin A8-like protein 1 | 0.148 | Down | 0.0049 | ANXA8L1 | 36.879 | 16.32 | 32.7 | 11 | 22 | 1 | 1.76 | 0.26 |
| P13928 | Annexin A8 | 0.133 | Down | 0.0124 | ANXA8 | 36.881 | 90.729 | 40.4 | 12 | 22 | 2 | 1.783 | 0.237 |
