# Supplementary figures and images for: Targeting nicotinamide N-methyltransferase overcomes resistance to EGFR-TKI in non-small cell lung cancer cells
Source: Cell Death Discov. 2022 Apr 6;8:170. doi: 10.1038/s41420-022-00966-x (PMC8986855; doi:10.1038/s41420-022-00966-x)

Unprocessed western blots


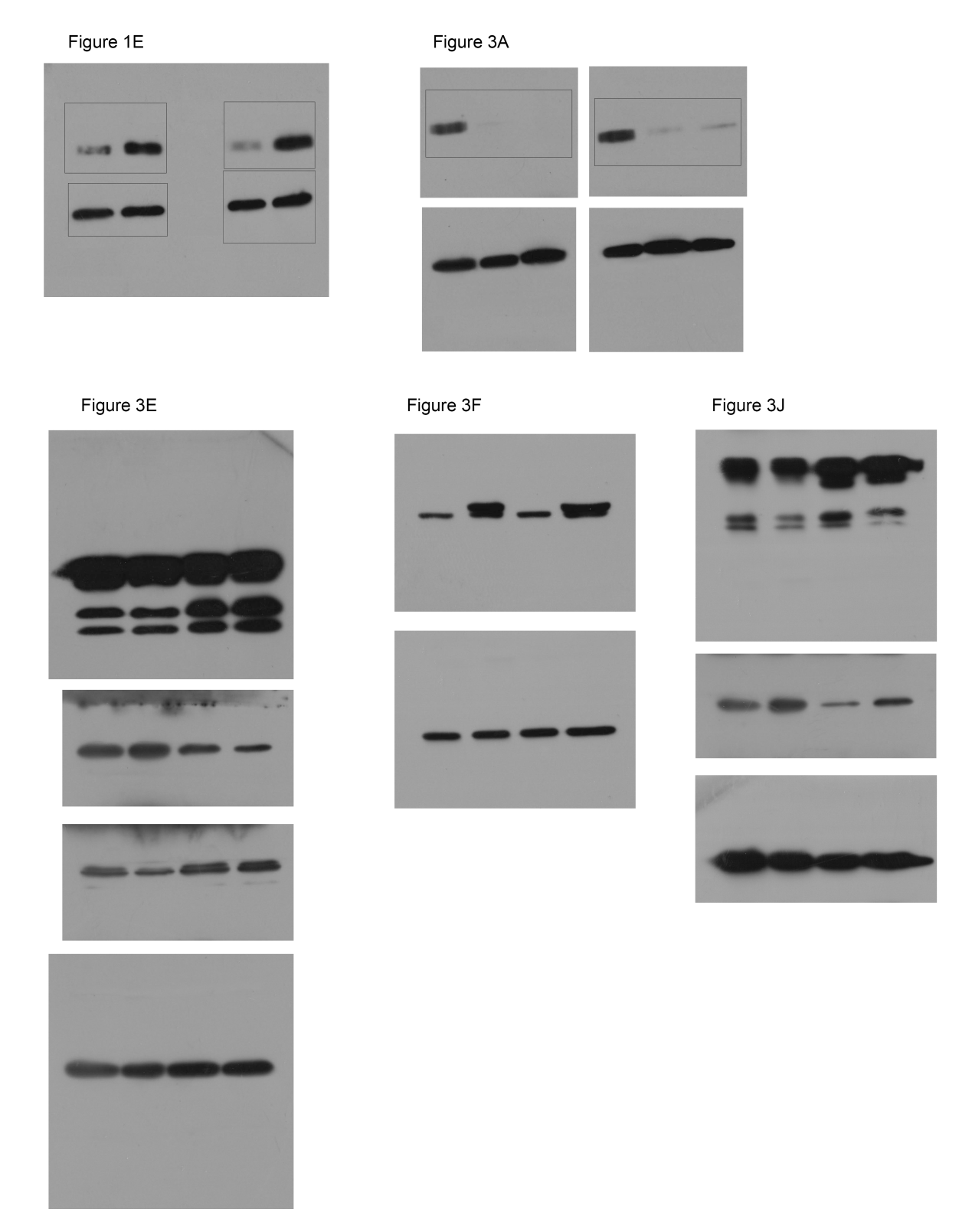


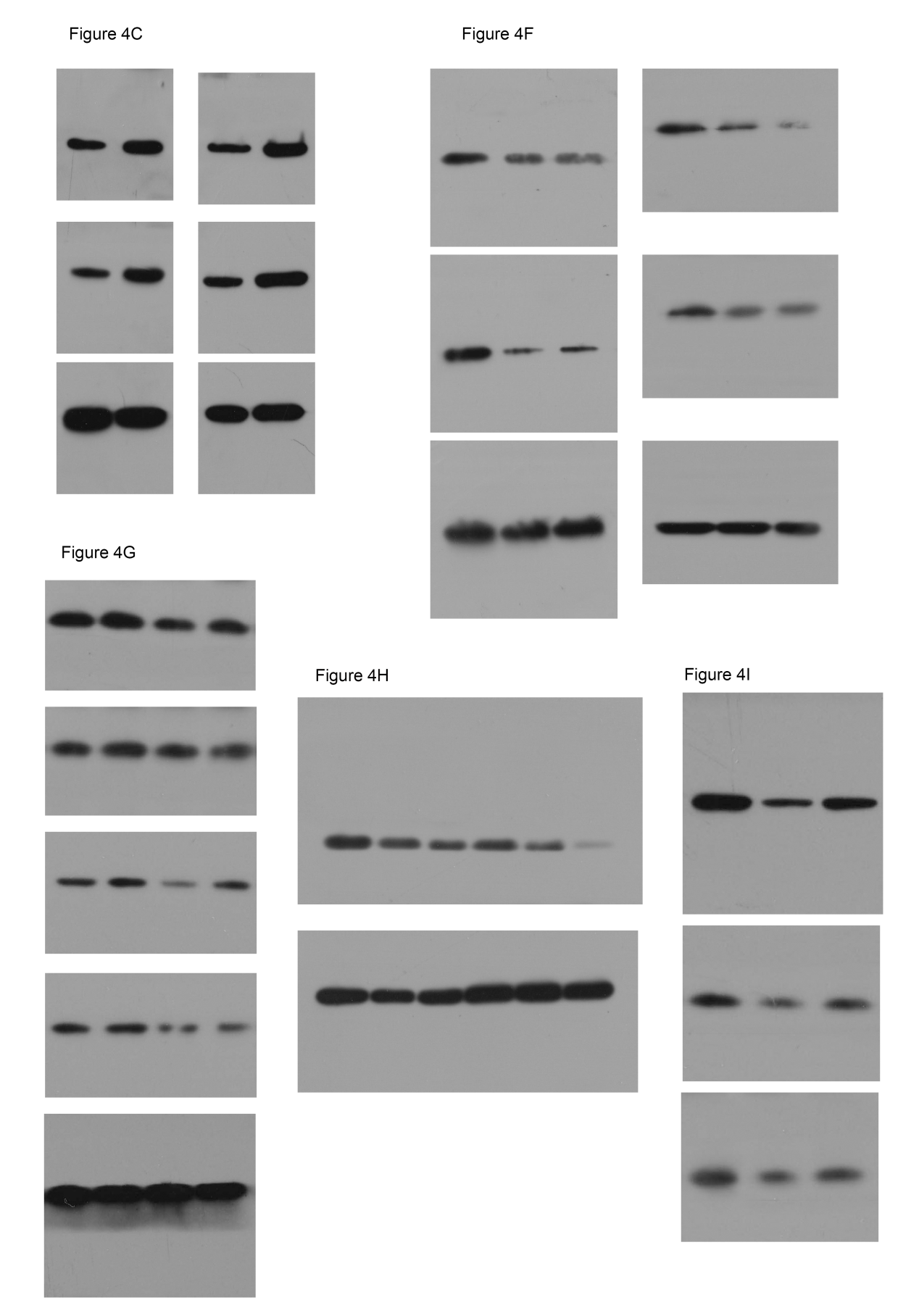


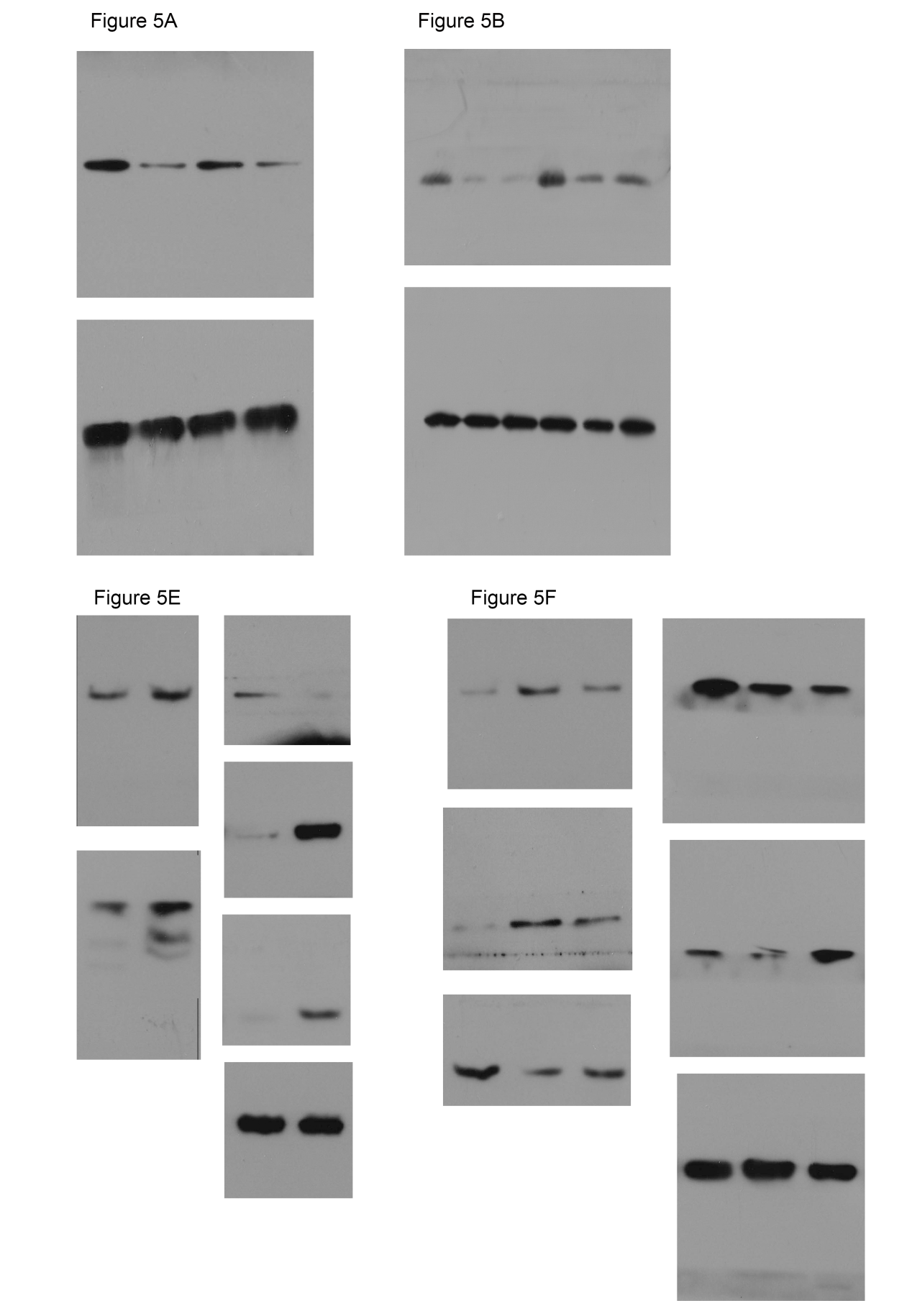

Supplement: Supplementary file 2 — Unprocessed western blots [file 41420_2022_966_MOESM2_ESM.docx]
